# The phageome of patients with ulcerative colitis treated with donor fecal microbiota reveals markers associated with disease remission

**DOI:** 10.1038/s41467-024-53454-4

**Published:** 2024-10-17

**Authors:** Marwan E. Majzoub, Sudarshan Paramsothy, Craig Haifer, Rohit Parthasarathy, Thomas J. Borody, Rupert W. Leong, Michael A. Kamm, Nadeem O. Kaakoush

**Affiliations:** 1 School of Biomedical Sciences, Faculty of Medicine and Health, UNSW, Sydney, Australia; 2 Concord Clinical School, University of Sydney, Sydney, Australia; 3 Department of Gastroenterology, Concord Repatriation General Hospital, Sydney, Australia; 4 School of Clinical Medicine, Faculty of Medicine and Health, UNSW, Sydney, Australia; 5 Department of Gastroenterology, St Vincent’s Hospital, Sydney, Australia; 6 Centre for Digestive Diseases, Sydney, Australia; 7 Department of Gastroenterology, St Vincent’s Hospital, Melbourne, Australia; 8 Department of Medicine, University of Melbourne, Melbourne, Australia

**Keywords:** Bacteriophages, Metagenomics, Ulcerative colitis

## Abstract

Bacteriophages are influential within the human gut microbiota, yet they remain understudied relative to bacteria. This is a limitation of studies on fecal microbiota transplantation (FMT) where bacteriophages likely influence outcome. Here, using metagenomics, we profile phage populations - the phageome - in individuals recruited into two double-blind randomized trials of FMT in ulcerative colitis. We leverage the trial designs to observe that phage populations behave similarly to bacterial populations, showing temporal stability in health, dysbiosis in active disease, modulation by antibiotic treatment and by FMT. We identify a donor bacteriophage putatively associated with disease remission, which on genomic analysis was found integrated in a bacterium classified to *Oscillospiraceae*, previously isolated from a centenarian and predicted to produce vitamin B complex except B12. Our study provides an in-depth assessment of phage populations during different states and suggests that bacteriophage tracking has utility in identifying determinants of disease activity and resolution.

## Abstract (web summary)

Here, the authors profile the gut phageome of individuals recruited into two double-blind randomized trials of Fecal Microbial Transplantation for ulcerative colitis, showing that phage communities are stable in health, dysbiotic in ulcerative colitis, modulated by antibiotics and by fecal transplants, with one *Oscillospiraceae* phage being associated with disease remission.

## Introduction

The human gut virome is largely made up of bacteriophages^1^, which are collectively referred to as the human gut phageome. It is most dense in the large intestine^2^ where bacterial density is also at its highest. Yet, despite phageome abundance^3^ and its important role in controlling bacterial diversity within the microbiota^4^, gaps remain in our understanding of the human gut phageome.

The gut phageome has been reported to be stable in health^5–7^. A range of factors do intersect to influence its composition, and these include age, diet, ethnicity, and geography (reviewed in Tiamani et al. ^4^). Among these factors is disease, with dysbiosis of the phageome being reported in a range of gastrointestinal diseases^4^, including the inflammatory bowel disease, ulcerative colitis (UC)^8,9^.

While UC is predominantly treated with immunological therapies that inhibit inflammation, direct manipulation of the microbiota in patients with UC has seen some level of success. Specifically, fecal microbiota transplantation (FMT), the transfer of healthy donor microbiota into a recipient, has been trialed as a therapeutic strategy, with efficacy rates ranging from 30–50%^10–14^. The majority of studies examining the influence of FMT in patients with UC has focused on the bacterial component of the microbiota, identifying a range of bacterial features associated with efficacy^15^. One study on a small cohort of patients with UC receiving FMT examining the virome suggested that there were no differences in the phageome of patients and donors, as opposed to eukaryotic viruses which were lower in donors when compared to patients and lower in responders prior to FMT when compared to non-responders^16^. In contrast, another study showed that patients with UC that responded to FMT had less phages compared to non-responders, and levels of bacteriophages correlated with mucosal IFN-γ^17^. In mice, the authors showed that bacteriophage expansion exacerbated colitis through the DNA sensing receptor TLR9^17^.

Research on the phageome is more consistent in FMT studies on recurrent *Clostridioides difficile* infection (rCDI). Allogenic FMT has been reported to shift patient virome composition towards the donor profiles^18,19^. Perhaps the most robust link for a role for the phageome in FMT outcomes in rCDI is a study that transferred sterile donor fecal filtrates, comprising a mixture of bacteriophages, into five patients with rCDI, showing restoration of normal stool habits for at least 6 months^20^. A role for the phageome in FMT outcome is further supported by the finding that rCDI cure was associated with increased presence of donor Caudovirales in patients following FMT^21,22^. Studies on metabolic syndrome also support a potential role for the phageome where allogenic FMT altered the gut phageome of recipients towards the donor profile^23^, and in a double-blind randomized placebo-controlled trial, administration of sterile fecal filtrates in patients with metabolic syndrome had a short-lived effect on recipient virome and led to a nominal improvement in glucose variability^24^.

The inconsistent findings on the phageome in UC despite the established importance of the phageome in modulating bacterial diversity and in contributing to outcomes of FMT in rCDI, emphasize the need for further well-controlled studies that examine bacteriophage populations in active UC, following FMT and in disease remission induced by FMT. Thus, here, we analyzed the phageome of patients and donors recruited to two independent randomized clinical trials of FMT in UC using shotgun metagenomics on fecal DNA extracts without prior virus-like particle (VLP) purification. Through assessment of patients and donors at different timepoints in the trials, we were able to study bacteriophage populations in clinically validated healthy individuals and contrast this with active disease. We were also able to determine the impact of antibiotic treatment and FMT on bacteriophage diversity and composition, as well as identify putative determinants of disease remission.

## Results

### Profiling the phageome in patients and donors across two independent clinical trials

We analyzed shotgun metagenomic data from two independent clinical trials of FMT to treat patients with UC to study the impact on fecal bacteriophage populations (phageome) as well as their association with response. In the first data set from the FOCUS trial, patients with UC at baseline (Tx0), week 4 of FMT (Tx4), week 8 of FMT (Tx8) and final follow-up 8 weeks after FMT treatment had concluded (TxF) as well as their donors (both donor batches and the individual donors contributing to the batches) were profiled. Patients who initially went on placebo therapy prior to open-label FMT were also profiled at the end of 8 weeks of placebo (P8). The second data set derived from the LOTUS trial included samples from patients with UC undergoing FMT at baseline, after antibiotics, at week 1 FMT, week 2, week 3, week 4 and week 8 (induction stage). It also included sampling during the maintenance phase for patients that were given low-dose FMT or those in the withdrawal arm. To complement the extended timeline of the LOTUS trial, longitudinal sampling of the two individual donors that participated in the trial was included. Analysis of the FOCUS data set identified 7,066 vOTUs, of which 2737 were taxonomically classified as a virus (as opposed to unknown). 113,271 vOTUs were identified in the LOTUS data, of which 25,941 were taxonomically classified as a virus. The variation in vOTU detection was likely a consequence of LOTUS samples being sequenced at a depth substantially greater than FOCUS samples (average read depth ± SEM: FOCUS: 3415827 ± 37684; LOTUS: 36799073 ± 1186931), and this was a key reason why the data sets were examined independently.

### Quality assessment of phageome profiles in the data sets

**Fig. 1:**
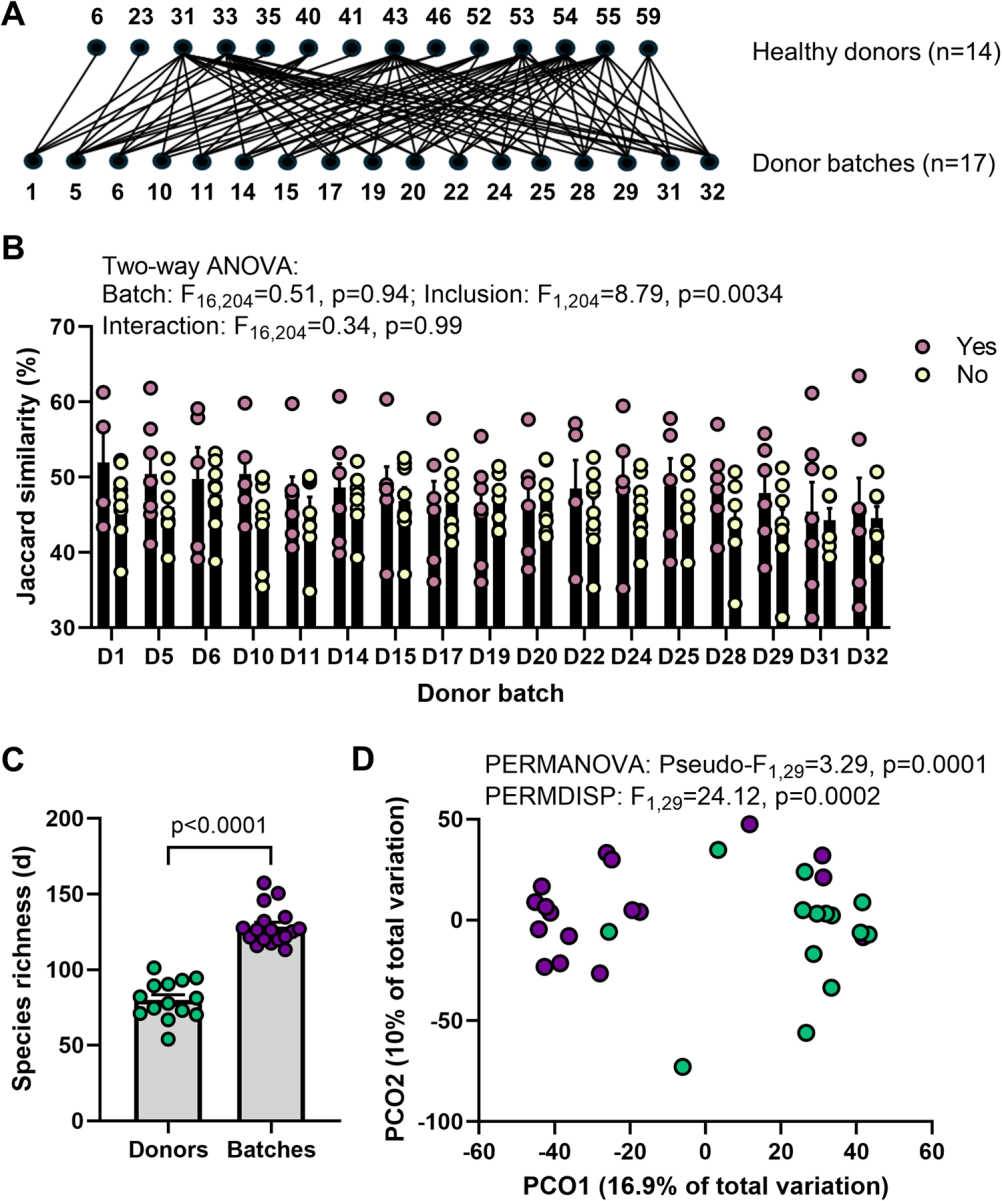
Quality assessment of phage identification. **A** The FOCUS trial design which included the combination of 4–7 individual donors into donor batches was employed to determine the accuracy of the phage mining strategy used. Only FOCUS vOTUs that were classified as a virus (*n* = 2737) were included in the analysis. **B** Jaccard similarity of the donor batches (D1, D5, D6, D10, D11, D14, D15, D17, D19, D20, D22, D24, D25, D28, D29, D31, D32) to the individual donors included (Yes, pink; *n* = 4, 7, 5, 5, 6, 6, 6, 6, 7, 6, 5, 5, 5, 6, 6, 7, 7, respectively) and not included (No, yellow; *n* = 10, 7, 9, 9, 8, 8, 8, 8, 7, 8, 9, 9, 9, 8, 8, 7, 7, respectively) in the batch. Differences were tested using two-way ANOVA with Batch x Inclusion as variables. Errors are ± SEM. **C** Margalef’s (species) richness in the individual donors (*n* = 14) and batches (*n* = 17). Differences were tested using a two-sided Mann-Whitney test (*p* < 0.0001) following assessment of normal distribution with Shapiro–Wilk test (Donors: *p* = 0.9537, Batches: *p* = 0.0293). Errors are ± SEM. **D** Principal coordinate analysis (PCO) on Aitchinson distances between individual donor (green) and donor batch samples (purple). Differences between groups were tested using PERMANOVA and PERMDISP. Source data are provided as a Source Data file.

We performed several quality assessments of the data and phage-detection method by leveraging the study design of the FOCUS clinical trial. Specifically, we compared the individual donors to the donor batches that they had contributed to (Fig. 1A). Our main assessment was whether the individual donors included within a batch were more similar to the batch than individual donors that were not included. Here, we employed Jaccard similarities (presence/absence of vOTU) rather than a metric that relies on relative abundance, to eliminate contribution bias from dominant phages. We found that individuals included in a batch were significantly more similar to the batch than those that were not included (*p* = 0.0034; Fig. 1B). We also identified that particular individual donors can often exhibit dominance in their contribution to the batch profile (top pink dots in Fig. 1B with substantially higher similarity). The results were consistent in the full dataset of 7,066 vOTUs (classified and unclassified) (Supplementary Fig. 1A). As an additional quality check, we also assessed the alpha and beta diversity metrics where it is expected that batches would be compositionally different as well as richer in vOTUs and more even. We find that the phageomes of donor batches were significantly more rich, more even and compositionally distinct when compared to the individual donors contributing to them (Fig. 1C,D; Supplementary Fig. 1B,C). These findings indicated that phage profiling in these samples was successful.

### The phageome in healthy individuals is stable across time

**Fig. 2:**
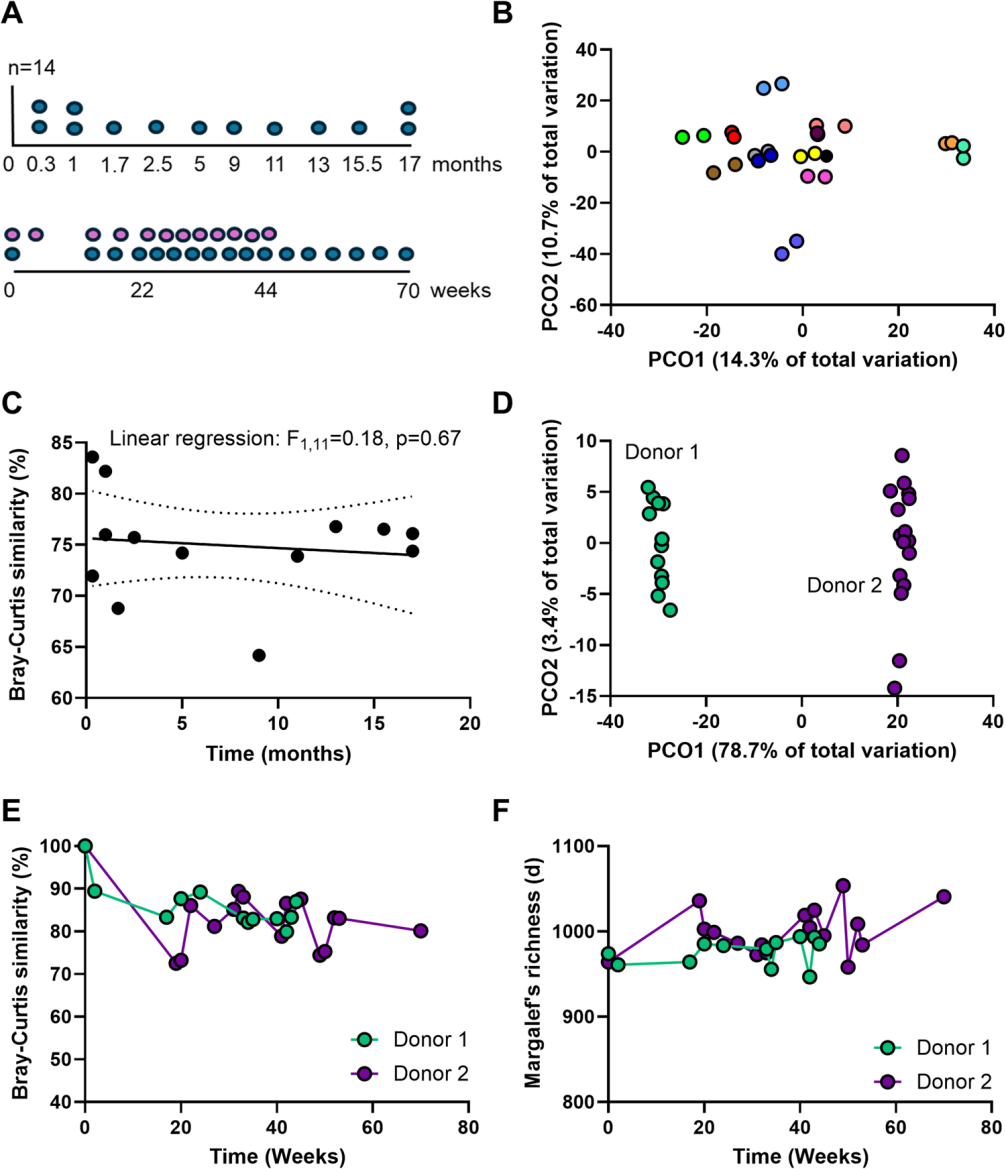
Phage diversity in healthy individuals. Donors from both clinical trials represent individuals that have been clinically validated as healthy. **A** Sampling from the donors in the FOCUS trial (top) and LOTUS trial (bottom). 14 donors in FOCUS were included and sampled at their baseline (0 months). 13 of 14 were sampled a second time over a period ranging from 0.3 to 17 months (blue points). Two donors in LOTUS were included that were sampled over 44 weeks (donor 1, *n* = 12, pink) and 70 weeks (donor 2, *n* = 17, blue). **B** Principal coordinate analysis (PCO) on Bray–Curtis similarities between samples from the individual donors. Only FOCUS vOTUs that were classified as a virus (*n* = 2737) were included in the analysis. **C** Linear regression between Bray-Curtis similarities intra-donor against time (months). The center line is linear regression equation and broken lines represent 95% confidence intervals. **D** PCO on Bray–Curtis similarities between samples from the two donors in LOTUS. The majority of the variation is observed inter-donor (PCO1 axis). Only LOTUS vOTUs that were classified as a virus (*n* = 25941) were included in the analysis. **E** Bray–Curtis similarities between samples from each donor in LOTUS over time. Values represent similarity with the sample prior in time with the first sample set at 100%. **F** Changes in Margalef’s (species) richness in samples from each donor in LOTUS over time. Source data are provided as a Source Data file.

Next, we examined donors in both clinical trials to establish the behavior of phage populations in healthy individuals over time. A highlight of our analysis is that these individuals represent a healthy status that has been validated by extensive clinical testing to pass the high threshold set for FMT donors. Of the 14 donors in the FOCUS trial, we had an additional sample from 13 donors that ranged from 0.3–17 months, while in the LOTUS trial, we had longitudinally sampled the two donors for 44 and 70 weeks (Fig. 2A). Analysis of the composition of the phage population across time in the FOCUS donors revealed a striking intra-donor similarity (Fig. 2B), that was independent of the time of sampling (Fig. 2C). This was confirmed by applying the same analysis on the full data set of classified and unclassified vOTUs (Supplementary Fig. 2A,B). While most repeat samples from the donors showed 70-80% similarity in phage composition intra-donor over time, it is worth noting that two donors showed higher than average similarity (>80%) whereas another two fell below average (<70%) (Fig. 2C).

In the two LOTUS donors, intra-donor variation was minimal (accounting for 3.4% of variation – PCO2) when compared to inter-donor variation (accounting for 78.7% of variation – PCO1) (Fig. 2D). This was supported by the high similarity between each donor and the sample preceding it (Fig. 2E) as well as the stability in phage richness over time (Fig. 2F). Thus, phage populations in individuals that were clinically confirmed to be healthy are stable over time and would serve as robust controls to compare against patients with disease.

### Patients with UC have a distinct phage population when compared to healthy controls

**Fig. 3:**
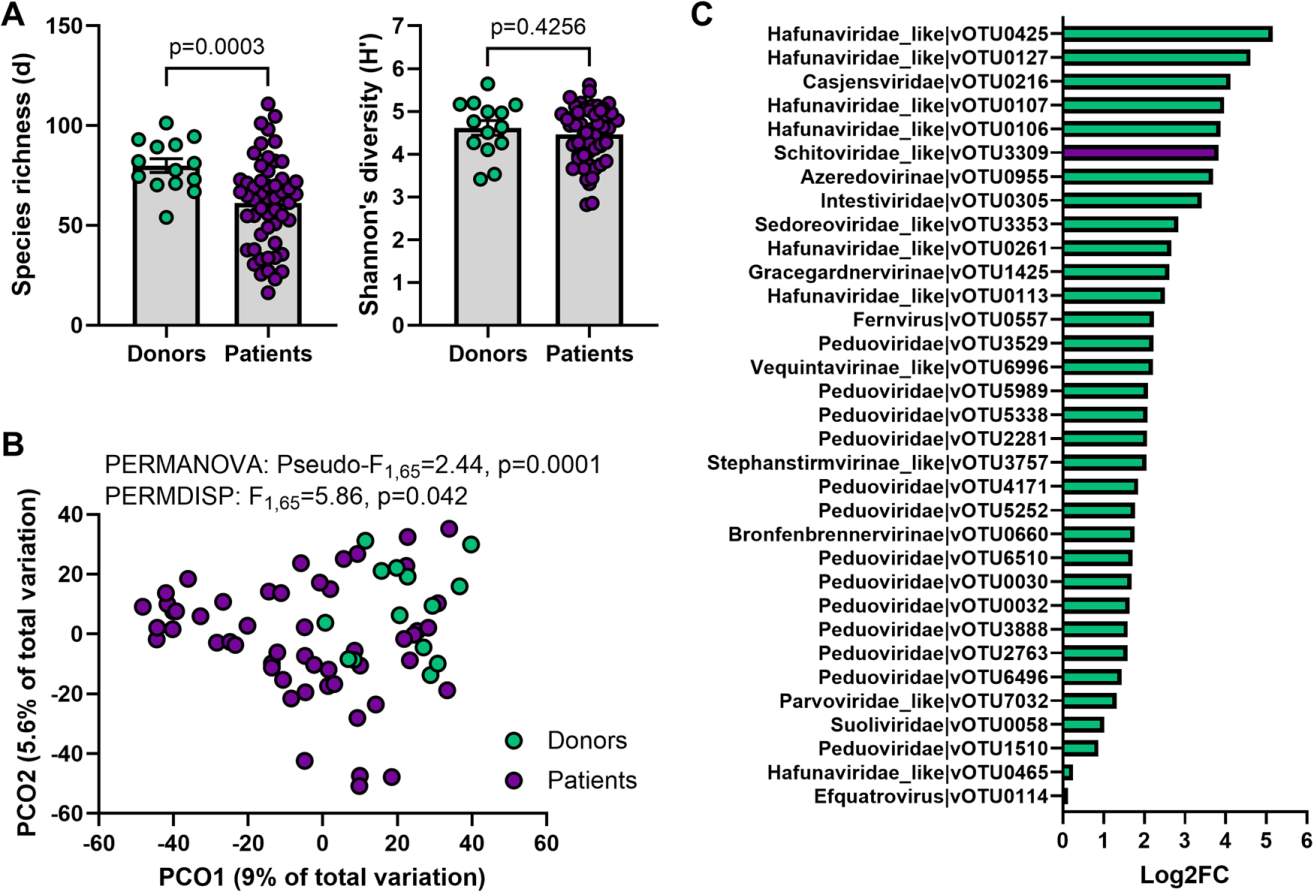
Phage diversity in active ulcerative colitis as compared to healthy individuals. FOCUS vOTUs that were classified as a virus (*n* = 2737) were included in the analysis. **A** Alpha diversity metrics (Margalef’s richness and Shannon’s diversity index) between donors (*n* = 14) and patients (*n* = 53). Differences in richness were tested using a two-sided Welch’s t-test (*p* = 0.0003) following assessment of normal distribution with Shapiro–Wilk test (Donors: *p* = 0.9537, Patients: *p* = 0.5537) and variances with F test (*p* = 0.0318). Differences in Shannon’s diversity were tested using a two-sided unpaired t-test (*p* = 0.4256) following assessment of normal distribution with Shapiro-Wilk test (Donors: *p* = 0.6160, Patients: *p* = 0.061) and variances with F test (*p* > 0.05). Errors are ± SEM. **B** Principal coordinate analysis (PCO) on Aitchinson distances between individual donors (green) and patient with active disease (purple). Differences between groups were tested using PERMANOVA and PERMDISP. **C** vOTUs that were identified to be significantly differentially abundant (*q* < 0.05) between donors and patients using ZicoSeq. Log2 fold-change (FC) was calculated manually for vOTUs enriched in donors (green) and patients (purple). Source data are provided as a Source Data file.

We then compared the donors in FOCUS with the patients recruited to the trial to determine if phage populations differed in UC. We found that patients at baseline (prior to FMT) had lower phage richness (*p* = 0.0003) but not Shannon’s diversity index (*p* = 0.42) when compared to controls (Fig. 3A). The composition of the phage population was also different to that of controls (*p* = 0.0001; Fig. 3B); however, a portion of this difference can be attributed to differences in dispersion between the two groups (*p* = 0.042), with phage profiles showing more variation inter-patient than inter-donor (Fig. 3B; Supplementary Fig. 3A). Analysis of differences at the vOTU level identified 33 vOTUs to be differentially abundant between patients and donors, with the majority (*n* = 32) being enriched in the donors (Fig. 3C). While statistically significant, caution should be taken in the interpretation of vOTUs with <2 Log2FC (*n* = 14), particularly vOTU465 and vOTU114. The only vOTU enriched in the patients (vOTU_3309; Log2Fold Change=3.82) showed 99.63% identity (99% query coverage) to several *Escherichia coli* genomes.

### Antibiotic treatment depletes the phageome

**Fig. 4:**
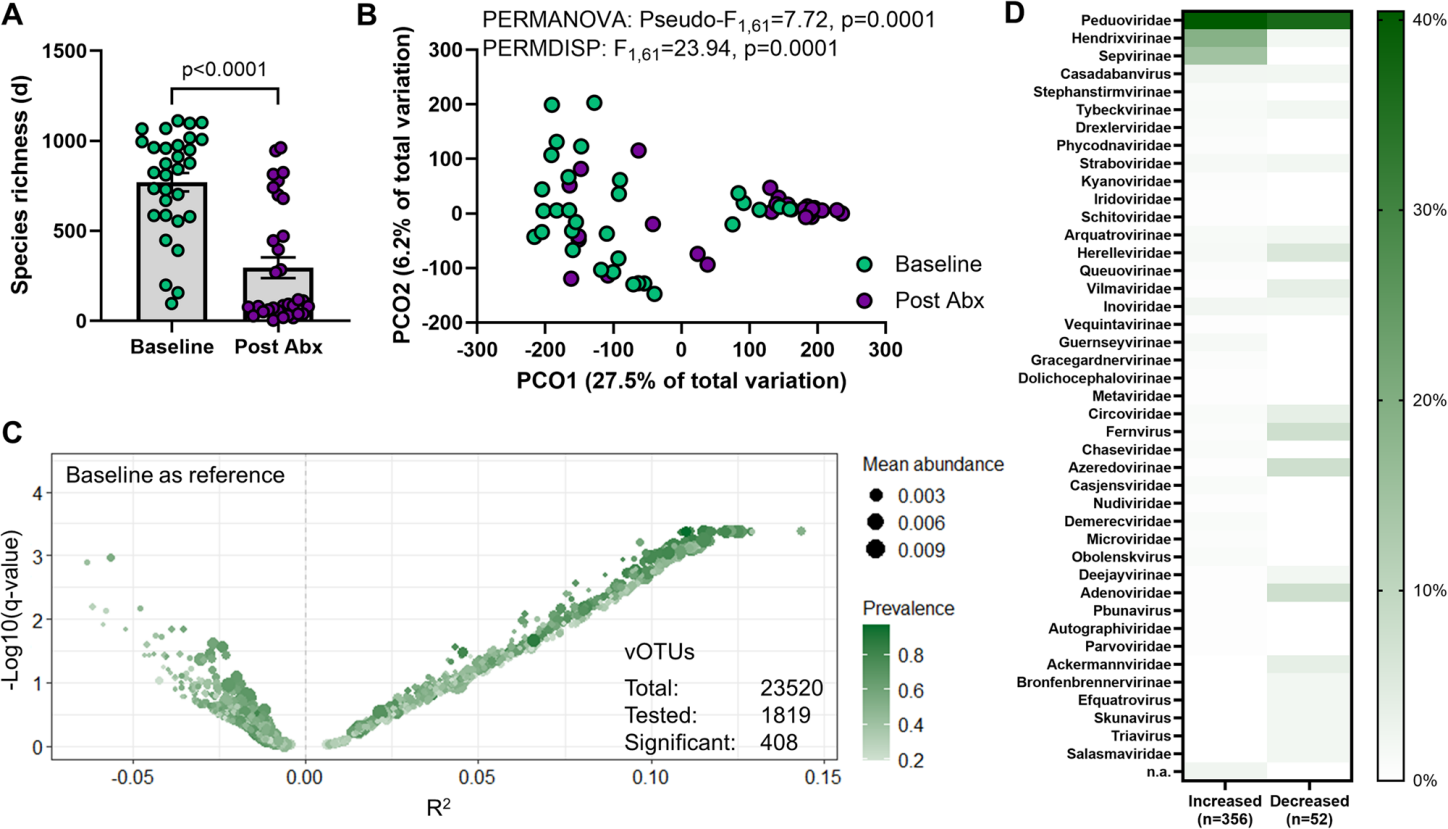
Effect of antibiotic treatment on phage diversity. LOTUS vOTUs that were classified as a virus (*n* = 25941) were included in the analysis. **A** Margalef’s (species) richness in patients at baseline (Baseline, *n* = 31) and patients following antibiotic treatment (Post Abx, *n* = 32). Differences were tested using a two-sided Mann–Whitney test (*p* < 0.0001) following assessment of normal distribution with Shapiro-Wilk test (Baseline: *p* = 0.0102, Batches: *p* < 0.0001). Errors are ± SEM. **B** Principal coordinate analysis (PCO) on Aitchinson distances between patients at baseline (Baseline, green) and patients following antibiotic treatment (Post Abx, purple). Differences between groups were tested using PERMANOVA and PERMDISP. **C** Volcano plot of differential abundance analysis of vOTUs between patients at baseline (Baseline) and patients following antibiotic treatment using ZicoSeq. 408 vOTUs were identified to be significantly differentially abundant between groups. **D** Classification of vOTUs that were significantly differentially abundant. Legend is % prevalence. Source data are provided as a Source Data file.

The LOTUS trial included antibiotic pre-treatment of patients to improve engraftment of donor species with FMT. We leveraged this design to establish the impact of a two week triple-regimen antibiotic therapy (amoxicillin, metronidazole, and doxycycline) on the phage population of patients. We identified a decrease in phage richness following antibiotics (Fig. 4A), mirroring the decreased richness in bacterial species^25^. We also observed a change in phage population composition with antibiotic treatment (Fig. 4B, Supplementary Fig. 3B), which was the result of 408 vOTUs being differentially abundant (Fig. 4C). However, contrary to expectations, the majority of these vOTUs (*n* = 356), mostly classified to *Peduoviridae*, *Hendrixvirinae* and *Sepvirinae*, were increased in relative abundance following antibiotics (Fig. 4D), possibly due to increased relative abundance of *Enterobacteriaceae*. Of note, several vOTUs found to be differentially abundant following antibiotic treatment were classified to eukaryotic DNA viral families including *Adenoviridae*, *Circoviridae* and *Parvoviridae* (Fig. 4D).

### Fecal transplants modify the gut phageome in patients with UC

**Fig. 5:**
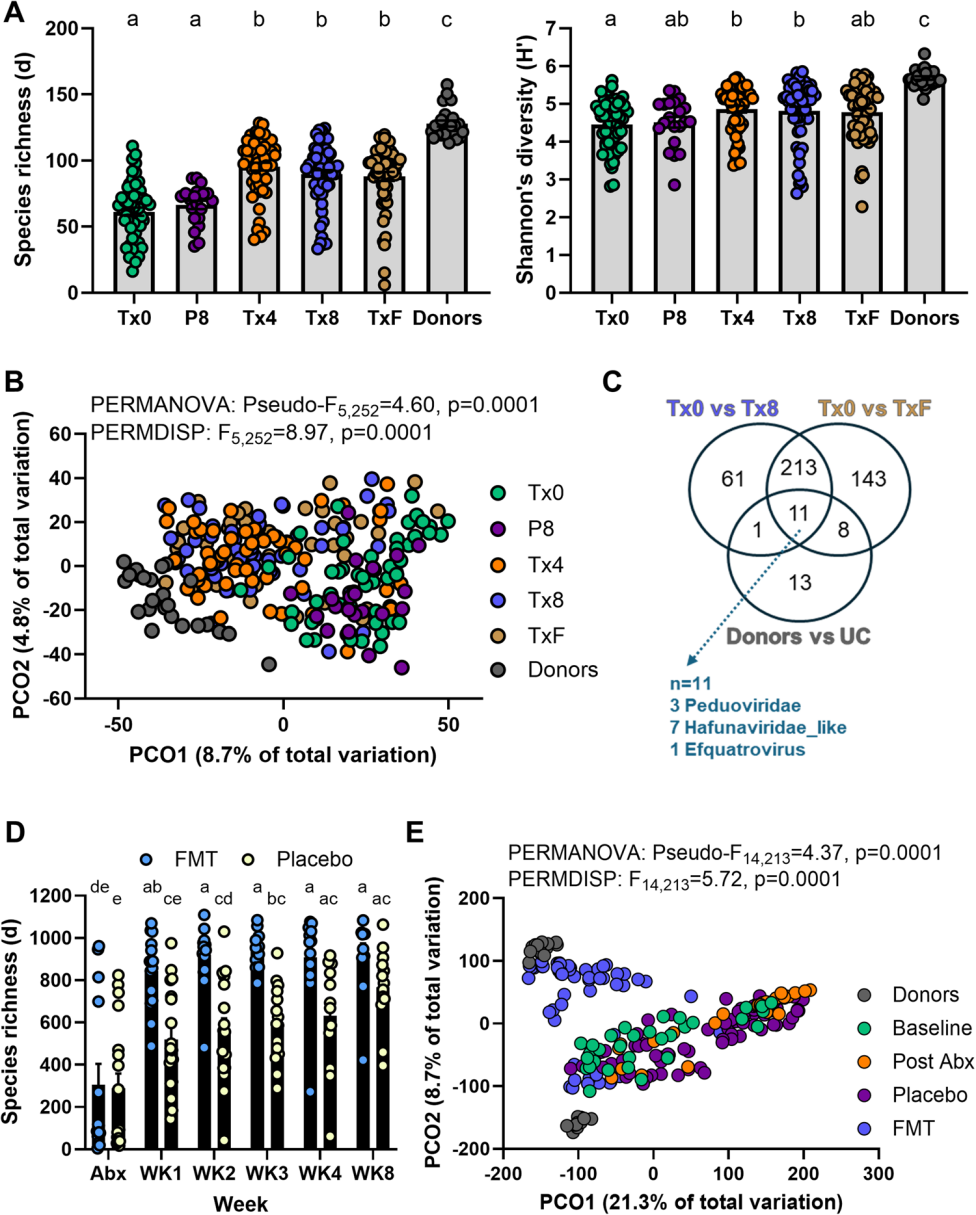
Effect of fecal microbiota transplantation on phage diversity. FOCUS and LOTUS vOTUs that were classified as a virus (*n* = 2737 and *n* = 25941, respectively) were included in the analysis where applicable. **A** Margalef’s (species) richness and Shannon’s diversity index of FOCUS donor batches (Donors, *n* = 17), patients at baseline (Tx0, *n* = 53), post 8 weeks of placebo (P8, *n* = 21), 4 weeks of FMT (Tx4, *n* = 53), 8 weeks of FMT (Tx8, *n* = 53) and at follow-up 8 weeks following FMT (TxF, *n* = 53). Differences for both metrics were tested using a Kruskal–Wallis test with a Dunn’s multiple comparisons test following assessment of normal distribution with Shapiro–Wilk test. Letters represent statistical significance. Errors are ± SEM. **B** Principal coordinate analysis (PCO) on Aitchinson distances for FOCUS samples across all groups. Differences between groups were tested using PERMANOVA and PERMDISP. **C** Venn diagram of significantly differentially abundant vOTUs identified by ZicoSeq across different comparisons in FOCUS samples (FMT: Tx0 vs Tx8, Follow-up post FMT: Tx0 vs TxF, and disease: donors vs UC). **D** Margalef’s (species) richness across time during FMT (Abx, *n* = 14; WK1, *n* = 15; WK2, *n* = 13; WK3, *n* = 11; WK4, *n* = 12; WK8, *n* = 10) or placebo (Abx, *n* = 18; WK1, *n* = 18; WK2, *n* = 17; WK3, *n* = 15; WK4, *n* = 12; WK8, *n* = 13) in LOTUS. Differences were tested using two-way ANOVA with Treatment x Week as variables (Treatment: *p* < 0.0001, Week: *p* < 0.0001, Interaction: *p* = 0.0376). Šídák’s multiple comparisons test was applied to identify differences across groups and statistical significance was represented as letters. Errors are ± SEM. **E** Principal coordinate analysis (PCO) on Aitchinson distances for LOTUS samples across all groups. Differences between groups were tested using PERMANOVA and PERMDISP. Donors, *n* = 29 samples from 2 donors; Baseline, *n* = 31 patients; Post Abx, *n* = 32 patients; Placebo, *n* = 75 samples; FMT, *n* = 61 samples. Source data and *p*-values for panels **a** and **d** are provided as a Source Data file.

We assessed if fecal transplants could modify the gut phageome of patients by direct comparison against placebo treatment either with or without antibiotic pre-treatment. In the FOCUS trial (without antibiotics), FMT increased phage richness beyond that observed with placebo, but richness in patients receiving FMT did not reach the level of the donor batches (Fig. 5A). FMT, but not placebo, led to a significant shift in phage composition towards the donor profiles (Fig. 5B, Supplementary Fig. 4A). We then screened for the vOTUs contributing to this compositional shift and identified 286 to be differentially abundant on 8 weeks of FMT when compared to baseline, and of these 224 vOTUs were maintained in the patients at 8 weeks follow-up (Fig. 5C, Supplementary Fig. 5). Of the 224 vOTUs, 11 were clear markers that could even differentiate between the donors and patients (Fig. 5C). No phages were found to be differentially abundant following placebo (Supplementary Fig. 5A). Similarly, in the LOTUS trial (with antibiotics), FMT led to an increase in phage richness that was not observed in placebo in the early stages of the induction arm (weeks 1–3, Fig. 5D, Supplementary Fig. 4B); however, phage richness in patients treated with antibiotics and then placebo appeared to recover to baseline by week 8 (Fig. 5D, Supplementary Fig. 4B). FMT had a significant effect on phage composition, with material from one of the donors appearing to be highly effective at engrafting phages (Fig. 5E, Supplementary Fig. 4C). Placebo consistently did not result in any differentially abundant phages (Supplementary Fig. 6A), and the number of differentially abundant phages following FMT was more modest than in the FOCUS trial (*n* = 46, Supplementary Fig. 6B,C) likely due to the single donor design in LOTUS. The results confirm that FMT has a considerable impact on the phageome in patients, unlike placebo, and that phage contribution to treatment efficacy should be considered.

### Specific phages are associated with treatment response across two clinical trials

**Fig. 6:**
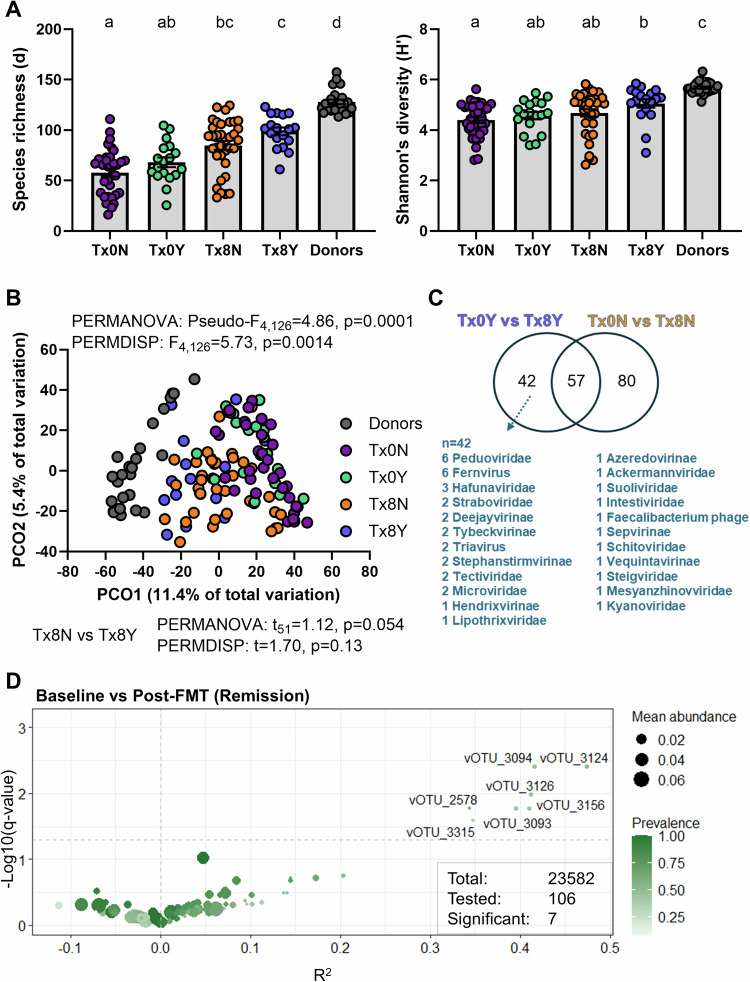
Phage diversity and response to fecal microbiota transplantation. FOCUS and LOTUS vOTUs that were classified as a virus (*n* = 2737 and *n* = 25941, respectively) were included in the analysis where applicable. **A** Margalef’s (species) richness and Shannon’s diversity index FOCUS donor batches (Donors, *n* = 17), non-responders at baseline (Tx0N, *n* = 35), responders at baseline (Tx0Y, *n* = 18), non-responders post FMT (Tx8N, *n* = 35) and responders post FMT (Tx8Y, *n* = 18). Differences for both metrics were tested using a Kruskal–Wallis test with a Dunn’s multiple comparisons test following assessment of normal distribution with Shapiro–Wilk test. Letters represent statistical significance. Errors are ± SEM. **B** Principal coordinate analysis (PCO) on Aitchinson distances for FOCUS samples across all groups. Differences between groups were tested using PERMANOVA and PERMDISP. Pairwise PERMANOVA and PERMDISP were performed to identify differences between Tx8N and Tx8Y. **C** Venn diagram of significantly differentially abundant vOTUs identified by ZicoSeq across different comparisons in FOCUS samples (Responders: Tx0Y vs Tx8Y, Non-responders: Tx0N vs Tx8N). **D** Differential abundance analysis of vOTUs of interest between patients at baseline (Baseline) and following FMT (Post-FMT) in the LOTUS dataset. Analysis with ZicoSeq was restricted to the 106 vOTUs that showed homology with the 42 vOTUs identified to be differentially abundant in responders in FOCUS. Source data and *p*-values for panel A are provided as a Source Data file.

We next determined if any aspects of the phageome was associated with response to FMT. We found that responders in FOCUS had a higher phage richness following FMT than non-responders, but this difference did not reach statistical significance (Fig. 6A). Phage composition showed a borderline non-significant difference between responders and non-responders following FMT (pairwise PERMANOVA *p* = 0.054, Fig. 6B, Supplementary Fig. 7A). Differences between responders and non-responders were clearer at the vOTU level, with a total of 179 vOTUs found to be differentially abundant, 57 shared between responders and non-responders, 80 unique to non-responders and 42 unique to responders (Fig. 6C). Results from LOTUS were more ambiguous, with non-responders showing a significantly increased phage richness following FMT that was not observed in responders (Supplementary Fig. 7B), and no clear patterns with phage composition (Supplementary Fig. 7C). This was likely due to the single donor design of the trial and the fact that non-responders were a more homogenous group (all receiving donor 2 with more potently engrafting phages (Fig. 5E)) while responders were mixed (some receiving donor 1 and others donor 2). Since the two donors in LOTUS were utilized in the donor batches of FOCUS, this presented a unique opportunity to identify vOTUs associated with response across the two trials. Sequences of the 42 vOTUs differentially abundant in responders in FOCUS were compared by blastn against the vOTU list of LOTUS, identifying 106 vOTUs that had >97% identity to them and a robust bit score. We selected these 106 vOTUs and assessed their association with response in the LOTUS trial, identifying seven vOTUs to be significantly enriched in responders (Fig. 6D). Of note, all seven vOTUs shared sequence similarity to 1 vOTU in FOCUS, vOTU_151 (total similar vOTUs to vOTU_151 was 11). While the behavior of phageome alpha and beta diversity metrics were not consistent across trials, our data revealed a group of related vOTUs to be associated with response to FMT during the induction period.

### Phages associated with response are further enriched in maintenance therapy

**Fig. 7:**
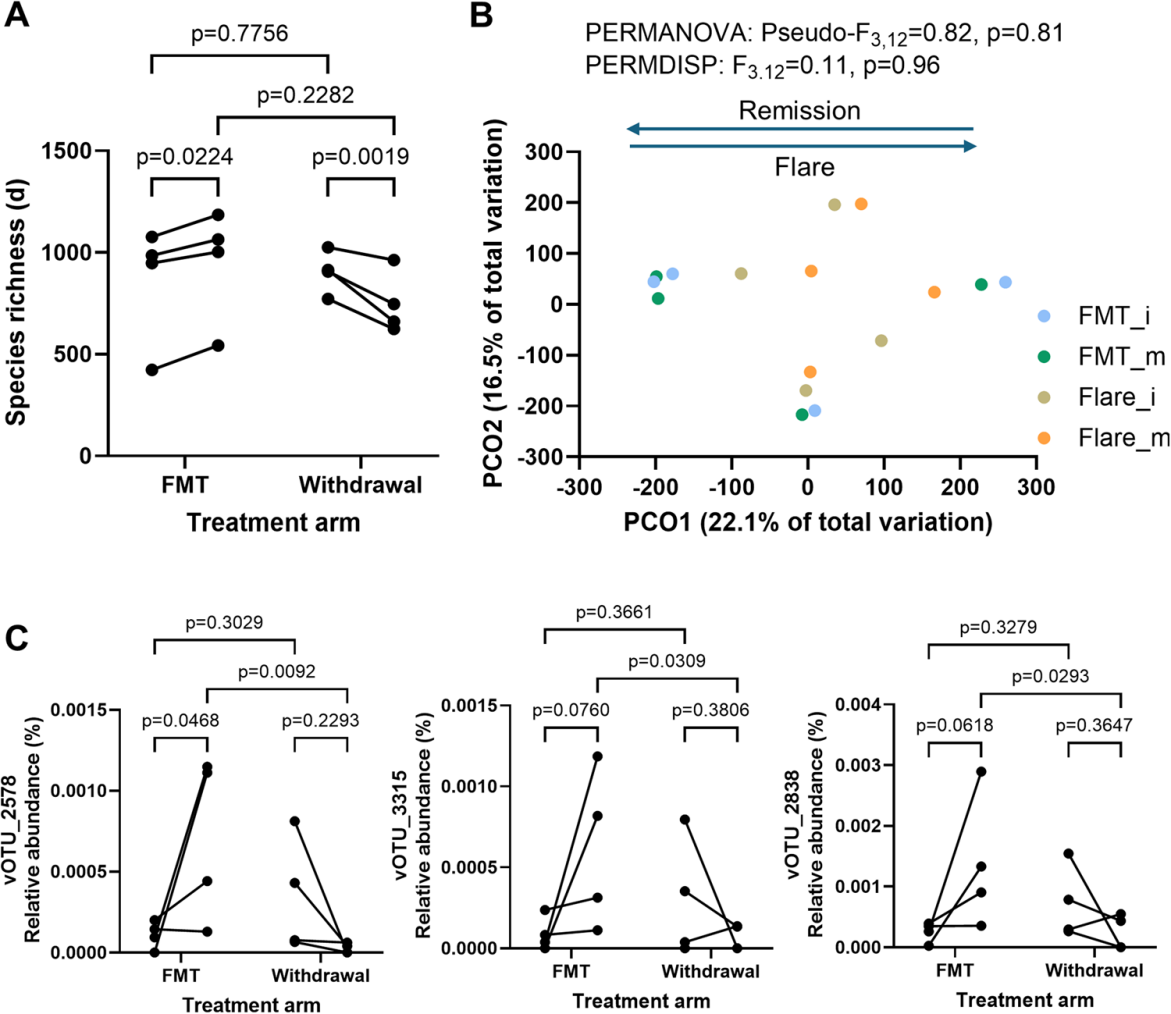
Phage diversity during maintenance therapy or withdrawal. LOTUS vOTUs that were classified as a virus (*n* = 25941) were included in the analysis. **A** Effect of low dose maintenance FMT (*n* = 4 patients) or withdrawal (flare, *n* = 4 patients) on Margalef’s (species) richness in the LOTUS trial. Differences were tested with a two-way repeated measures ANOVA and multiple comparisons testing was performed using an uncorrected Fisher’s LSD. **B** Principal coordinate analysis (PCO) on Aitchinson distances for LOTUS samples across all groups. Differences between groups were tested using PERMANOVA and PERMDISP. FMT_i: patients at end of induction FMT therapy that went into maintenance arm, FMT_m: patients at end of maintenance FMT therapy, Flare_i: patients at end of induction FMT therapy that went into withdrawal arm, Flare_m: patients who withdrew from therapy around the time of disease flare. **C** vOTUs that had significantly altered relative abundances during maintenance FMT or withdrawal (*n* = 4 patients sampled before and after for each group). Only LOTUS vOTUs with homology to vOTU_151 in FOCUS were tested. vOTU_2578 and vOTU_3315 were associated with response at induction. Differences were tested with a two-way repeated measures ANOVA and multiple comparisons testing was performed using an uncorrected Fisher’s LSD. Source data are provided as a Source Data file.

The LOTUS trial included a small maintenance arm that saw responders either be randomized to low dose FMT and maintain response (*n* = 4) or withdraw from therapy and flare within 6 months (*n* = 6 but only 4 had longitudinal fecal samples). Low dose maintenance FMT (2 capsules daily) was defined relative to the number of capsules patients received in the induction phase^25^. We assessed the impact this had on the phageome, and we found that consistent with the induction arm, maintenance FMT led to an increase in phage richness whereas withdrawal of FMT led to a decrease in richness (Fig. 7A). While patient numbers were low, we found a consistent pattern in phage composition on the PCO1 axis, where patients on maintenance FMT that remained in remission shifted in the opposite direction to those that withdrew from therapy and flared (Fig. 7B, Supplementary Fig. 8A). We then tested the changes to the 11 vOTUs with sequence similarity to vOTU_151, and found that while most displayed increases during low dose FMT and decreases with therapy withdrawal (Fig. 7C, Supplementary Fig. 8B,C), 3 (vOTU_2578, vOTU_3315, vOTU_2838) reached statistical significance (Fig. 7C). This suggested that the group of phages differentially abundant in responders in the induction arms of FOCUS and LOTUS, displayed relative abundance changes consistent with an association with remission in the maintenance arm. However, caution should be taken when interpreting these findings as the relative abundances of the vOTUs are low and multiple comparison corrections have not been performed.

### Phages associated with response are integrated into an *Oscillospiraceae* strain

**Fig. 8:**
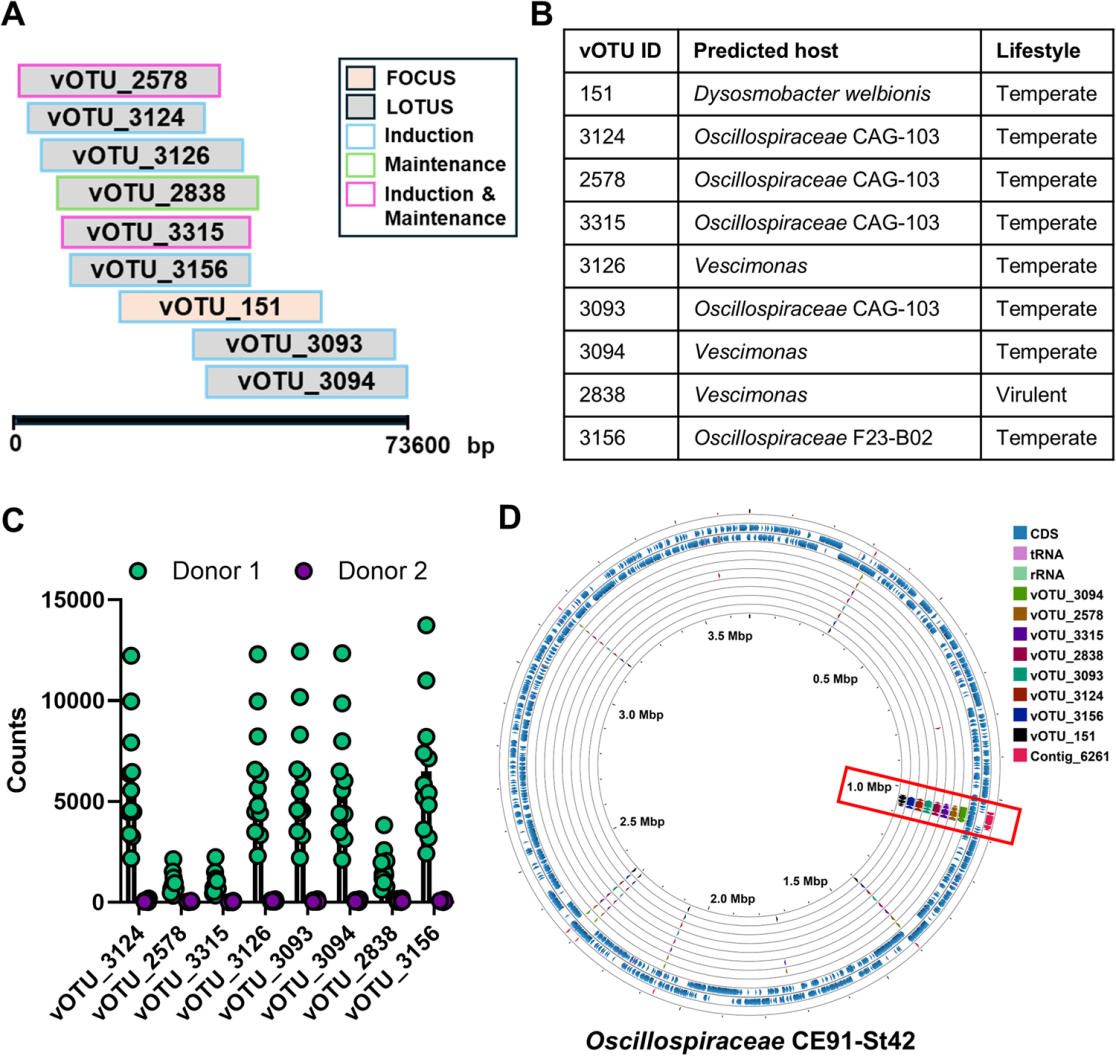
Identification of the bacterial host of phages associated with response to FMT. **A** Alignment of FOCUS and LOTUS vOTUs of interest and their association with response across the different analyzes. Alignment was performed using Clustal Omega and a simplified representative diagram is shown. **B** Predictions of the host and lifestyle of the vOTUs of interest. Predictions were made using ipHOP and PhaBox. **C** Comparison of the raw counts of the LOTUS vOTUs of interest between the two donors. Donor 1 (*n* = 12) had higher FMT efficacy that donor 2 (*n* = 17) in the LOTUS trial. **D** Alignment of vOTUs of interest and long-read contig of interest (Contig_6261) to the genome of *Oscillospiraceae* CE91-St42, isolated from a Japanese centenarian. Alignment and visualization were performed using Proksee. Source data are provided as a Source Data file.

We then explored the relevance of the associations between the vOTUs and response to FMT. We first examined their relationship with each other, identifying how the vOTUs aligned based on sequence similarity (Fig. 8A). We predicted their host and lifestyle, which revealed that all putatively targeted members within the family *Oscillospiraceae* and the majority (except 1) were predicted to be temperate (Fig. 8B). As these phages were donor-derived with the LOTUS vOTUs being enriched in donor 1 (the donor with 100% success) (Fig. 8C), and vOTU_151 (from FOCUS) being detected in the same individual donor in the FOCUS data, we explored long read sequencing of this donor as a method of validation. We identified within the long-read data several contigs (particularly contig_6261; Supplementary Table 1) that were identical to the vOTUs from the short-read sequencing, validating the presence of this phage in donor 1. Moreover, as the vOTUs were predicted to be temperate, we searched for possible integration into a bacterial genome to identify the culprit bacterium that would in turn be relevant to treatment response. We identified robust evidence that the vOTUs in question as well as the long-read contigs were all identical to a phage integrated to a previously isolated and sequenced bacterium from Japan, *Oscillospiraceae* bacterium CE91-St42 (Fig. 8D, Supplementary Table 1). To understand the potential importance of this bacterium, we created a gap-filled genome-scale metabolic model and a pathway enrichment analysis was performed. We identified enriched pathways related to vitamin B complex [KEGG map00770 (B5): *q* = 8.63E-13, map00740 (B2): *q* = 1.03E-09, map00730 (B1): *q* = 9.64E-09, map00750 (B6): *q* = 6.92E-07, map00760 (B3): *q* = 0.00045, map00790 (B9): *q* = 0.047, and map00780 (B7): *q* = 0.097] within the predicted metabolic output of this bacterium, and on further inspection, this bacterium was predicted to produce all vitamin B complex (B1, B2, B3, B5, B6, B7 and B9) except for B12.

## Discussion

To improve our understanding of the bacteriophage population in UC and in response to FMT treatment, we analyzed the phageome in individuals from two independent randomized clinical trials of FMT in UC. We identify that bacteriophage populations are stable in healthy individuals, altered in richness and composition in patients with active UC, can be modulated with antibiotics and FMT in these patients, as has been reported for the bacterial component of the microbiome. We found specific bacteriophages that were putatively associated with disease remission, and on further analysis, they represented a phage detected in the genome of a bacterial taxon classified to *Oscillospiraceae* and previously isolated from a Japanese centenarian^26^. This highlighted the importance of phage populations in the context of UC and microbiome manipulation as well as the ability of phage profiling to putatively identify bacterial taxa that could be determinants of response to FMT.

Given that methods to analyze bacteriophages are less established than those for bacteria, we initially assessed the data output from MetaPhage by using the donor design in the FOCUS trial to determine how well vOTU counts aligned with the biological combinations. Specifically, 14 individual donors were included into 17 donor batches in different combinations, with sequences available for all. We find that the phageome output is robust, in that donors contributing to a batch were significantly more similar to the batch than those that did not. All batches were also richer than any individual donor. An unexpected finding from the analysis was the dominance of particular donors to the batch composition and this was not always the same donor. This is an important outcome that in the future needs to be accounted for in the context of multi-donor FMT.

We characterized the phageome of our donors over time. The strength of this analysis is that these donors are validated as healthy through an extensive testing regimen^27^. We showed that the phage population is highly stable in these individuals over a long period of time, and this is in line with previous work in healthy individuals^5–7^. In our study, stability in health is shown in two different contexts. First, two samples taken from the same individual over one year apart are as similar to samples taken weeks apart. Second, over an extended period of time (up to 70 weeks), inter-donor difference was by far the biggest contributor to variability (78.7%) when compared to intra-donor difference (3.4%). Further, by comparing against these clinically validated healthy individuals, we were able to observe phageome dysbiosis in ulcerative colitis, where patients with active disease had lower phage richness and a shift in phage composition, marked by depletion of 32 vOTUs and enrichment of one vOTU that showed evidence of integration into *E. coli*. Therefore, phages may provide inferences into the dynamics and behavior of bacteria, since *E. coli* is known to be enriched in UC due to the colonization advantage provided by intestinal inflammation^28^.

We observed that the phageome in patients can be modified by antibiotic treatment. The impact of a triple-regimen antibiotic treatment on phage diversity in our patients, particularly the contraction of richness, is in line with a previous study that assessed the virome of patients receiving *Helicobacter pylori* eradication therapy (two antibiotics or more)^29^. However, our results differ in that Wang and colleagues reported impacts on the virome for up to 6 months^29^, whereas in our patients treated with antibiotics then receiving placebo, phage richness and composition did not differ significantly from baseline (pre-antibiotics) by 8 weeks. Among the vOTUs that were found to be differentially abundant with antibiotics were several that were classified to eukaryotic DNA viruses such as *Adenoviridae*, *Circoviridae* and *Parvoviridae*. The importance of this finding remains unclear but the presence of these viruses at baseline in patients with active UC should be explored further.

In our study, unlike what was previously reported^16^, FMT had a striking effect on phage richness and composition both when given with and without antibiotic pre-treatment. In keeping with the lower richness seen in patients with UC at baseline, FMT increased richness and shifted composition towards the donors. We speculate that in the FOCUS study this may be due to the transplant of supraphysiological levels of phages that exist within the donor batches (made up of several individual donors), whereas in the LOTUS study, this was the result of prior antibiotic depletion of phage population richness. Our findings are also in line with a recent study by Zuppi *et al* that explored the impact of FMT on the phageome of recipients being treated for obesity and comorbidities^30^. The authors reported donor phage engraftment leading to a shift in recipient phageome composition towards the donors and an increase in phage richness in females following FMT^30^.

When stratifying those that achieved the primary endpoint (steroid-free clinical remission with endoscopic remission or response) from those that did not, we did not observe consistent signatures in phageome alpha and beta diversity between the trials. For example, responders in FOCUS had higher phage richness and different composition than non-responders following FMT albeit not significant, whereas non-responders in LOTUS had a significant increase in richness that was not seen in responders. Notably, post-FMT richness did not differ between responders and non-responders in LOTUS despite the significant increase in non-responders. This may be a consequence of the differences in trial design including the antibiotic preconditioning, route of delivery, and single vs multi donor FMT. In contrast to diversity metrics, at the individual taxon level we identified vOTUs that were consistently enriched in responders across both trials, and these corresponded to a phage with a predicted bacterial host classified to *Oscillospiraceae*. Genomic analysis of this phage, including validation of its presence using long-read sequencing of the effective donor that harbors high levels of it, showed striking evidence of integration of this phage in a specific *Oscillospiraceae* strain isolated from a Japanese centenarian. The microbiome of this centenarian was reported to produce a unique profile of secondary bile acids with potency against gut pathobionts, but it did not appear that this strain contributed to this activity^26^. Given this, we generated a genome-scale metabolic model of this bacterium using the publicly available genome which determined that it was enriched in pathways related to vitamin B complex. Specifically, it was predicted to produce all vitamin B complex except for B12 with no required cooperation from other taxa. This is relevant to UC as vitamin B complex can influence production of beneficial short chain fatty acids by other members of the microbiota^31^, lower levels of vitamin B5 is a consistent feature in patients with IBD^32–35^, and meta-analysis has shown serum folate levels but not B12 were reduced in patients with UC^36^.

The study has several limitations. Our findings are from shotgun metagenomic sequencing with no prior VLP purification, meaning the data cannot differentiate between temperate phages and prophages. As a result of sequencing DNA, we attempted to limit our viral analysis to phages but several eukaryotic DNA viruses were also identified. Future work can consider optimized VLP purification and sequencing protocols that include exogenous phage spiking of fecal extracts for quantitative outputs^37^. We could not confirm the importance of levels of vitamin B complex in our patients as these measurements were not available. Future work should explore the relationship between this phage and levels of vitamin B complex. Moreover, the association between the taxon *Oscillospiraceae* bacterium CE91-St42 and its phage with clinical resolution following FMT remains putative and requires experimental validation to establish causality. *Oscillospiraceae* bacterium CE91-St42 was not identified to be associated with clinical resolution in past studies assessing the bacterial component of the microbiota^25,38–40^; however, this may have been a consequence of the isolate and its specific genome becoming available in 2022.

In conclusion, these findings from data across two double-blind, randomized, placebo-controlled trials identify a determinant of response to FMT treatment in UC by analyzing the phage populations of patients and donors. We have also shown that analyzing phage populations can identify otherwise overlooked candidates, including bacterial members of the microbiota, contributing to health and disease. Establishing the potential therapeutic benefits of supplementation with the bacterial species corresponding to *Oscillospiraceae* bacterium CE91-St42, or the temperate phage integrated within the bacterium, in patients with UC may prove to be an exciting outcome of this work. Future work could also consider the utility of these phage biomarkers for the selection of optimal FMT donors as well as testing the ability of the sterile stool filtrates from these donors to induce remission in patients with UC.

## Methods

### Ethics approval

The FOCUS investigator-initiated study was sponsored by the University of New South Wales, approved by the St Vincent’s Hospital Sydney Human Research Ethics Committee (HREC/13/SVH/69) and registered with ClinicalTrials.gov (NCT01896635) and the Australian Therapeutic Goods Administration Clinical Trial Notification Scheme (2013/0523). The LOTUS investigator-initiated study was approved by the St Vincent’s Hospital Sydney Human Research Ethics Committee (HREC/18/SVH/219) and registered with the Australian New Zealand Clinical Trial Registry ACTRN12619000611123. Written informed consent was obtained from all participants in both clinical trials prior to enrollment in the study, and this included consent for the metagenomic sequencing and analysis reported in this work.

### Study cohorts

Patients and donor stool samples from the FOCUS clinical trial (*n* = 285) were previously profiled using shotgun metagenomic sequencing on an Illumina HiSeq 2500 with 2 × 250 bp chemistry (DNA input of 1 ng) at the Ramaciotti Centre for Genomics^38,39^. The bacterial component of the gut microbiome in patients and donors was assessed previously^39^. At the time, total DNA was extracted from fecal samples using the MOBIO PowerViral RNA/DNA Isolation kit (Catalog no. 28000-BUNDLE) without prior VLP purification. When combining the blinded and open-label arms of the trial, samples include patients at baseline (Tx0, *n* = 53), the same patients at week 4 of FMT (Tx4, *n* = 53), the same patients at week 8 of FMT (Tx8, *n* = 53), the same patients 8 weeks after the conclusion of FMT treatment (TxF, *n* = 53), individual donors recruited to the trial (27 samples from *n* = 14 donors), and the multi-donor batches transplanted into the patients (25 samples from *n* = 17 batches). Samples were also collected from patients who initially received 8 weeks of placebo in the blinded arm (P8, *n* = 21) before transitioning to open-label FMT. The 53 patients (20 female) had a mean age ± SD of 37.50 ± 11.83 years.

Patients and donor stool samples from the LOTUS clinical trial (*n* = 250) were profiled using shotgun metagenomic sequencing^25,40^. Total DNA was extracted from the fecal samples using the QIAamp PowerFecal DNA Kit (Qiagen; Chadstone, Vic, AU). Sequencing of total DNA (no prior VLP purification) was performed at the Ramaciotti Centre for Genomics (UNSW Sydney) where DNA was prepared using Illumina DNA prep kits (Illumina; Melbourne, Vic, AU) and sequenced on an Illumina NovaSeq 6000 S4 run using 2x150bp chemistry (DNA input of 500 ng). Two negative controls were included that generated no data following library preparation and sequencing. In this trial, patients received antibiotics for 2 weeks and were then randomized to either single-donor FMT (*n* = 15) or placebo (*n* = 20) for 8 weeks, after which a subset of patients receiving FMT were randomized to a maintenance arm where they were administered low dose FMT or withdrew from therapy. Total samples included patients at baseline (*n* = 31), patients following antibiotics (*n* = 32), patients at week 1 (*n* = 33), week 2 (*n* = 30), week 3 (*n* = 26), week 4 (*n* = 24) or week 8 (*n* = 23) treatment, patients at week 20 (*n* = 5), week 32 (*n* = 9), week 44 (*n* = 3), week 56 (*n* = 5) treatment or withdrawal, donor 1 samples (*n* = 12), and donor 2 samples (*n* = 17). The bacterial component of the gut microbiome in the donors was assessed previously^40^. The 35 patients (17 female) had a mean age ± SD of 37.37 ± 11.68 years.

### Phage identification

Phage sequences in the shotgun metagenomes were identified using the MetaPhage standard operating procedures (SOP)^41^ with some modifications. Briefly, reads were assembled using MEGAHIT v1.2.9^42^ with default parameters and the quality of the scaffolds were evaluated using metaQuast v5.0.2^43^. Putative viral contigs were identified from the assembled scaffolds using five phage mining tools: DeepVirFinder (v1.0)^44^, VIBRANT (v1.2.0)^45^, Phigaro (v2.3.0)^46^, VirSorter2 (v2.2.3)^47^ and VirFinder (v1.1)^48^. Identified sequences were dereplicated using CD-HIT-EST (v4.8.1)^49^ and consensus viral contigs, now called viral operational taxonomic units (vOTUs), were assessed with CheckV (v 0.9.0)^50^ as per the MetaPhage SOP. The R script based on the UpSet R library (v1.4.0) available in the SOP^51^ was then used to compare results from different phage-mining tools and determine how many are mined by two or more tools. To generate a count table of the vOTUs, Bowtie2 (v2.4.2)^52^ quantified vOTUs in the preprocessed read sets by mapping them and processing outputs with SAMtools (v1.11)^53^, followed by count table construction using the bamcountrefs module from the BamToCov tool (v2.0.4)^54^.

### Phage annotation and taxonomic classification

To annotate and classify vOTUs, we first employed the tools suggested within the MetaPhage SOP. Specifically, Prodigal (v2.6.3)^55^ in metagenome mode enabled the prediction of open reading frames from the vOTUs, and these protein sequences were aligned to reference databases using DIAMOND (v0.9.14)^56^, to then be classified using vConTACT2 (v0.9.19)^57^. In addition to this, vOTUs were classified using PhaGCN1 and PhaGCN2^58^ based on the International Committee on Taxonomy of Viruses virus taxonomy profile (2021).

### Data analysis

Raw vOTU counts were used to calculate alpha diversity metrics (Margalef’s richness, Pielou’s evenness and Shannon’s diversity index) in Primer-e v6. All statistical analysis on these metrics, including testing for normal distribution using the Shapiro-Wilk test, were performed using GraphPad Prism v10. Beta diversity was assessed in two ways and analyzes were conducted in Primer-e v6 unless otherwise stated. First, raw vOTU counts were centre-log transformed using the Tjazi package (clr_lite option with 1000 replicates)^59^ in R v4.3.2 (RStudio 2023.12.1 Build 402), and this was followed by calculation of Euclidean distances between samples in Primer-e v6 – for the generation of Aitchinson distances. Distances were ordinated using Principal Coordinate Analysis (PCO) and differences between groups tested using permutational multivariate ANOVA (PERMANOVA) and analysis of dispersions (PERMDISP). To validate the above analysis, raw vOTU counts were normalized to relative abundances (%) and square root transformed. Bray-Curtis similarities were calculated on the transformed data and then ordinated using PCO. Inter-group differences were tested using PERMANOVA and PERMDISP. Differential abundance of specific vOTUs between groups was tested using ZicoSeq^60^ according to the following criteria: (1) filtration to remove rare taxa prev.filter=0.2 and max.abund.filter=0.002, (2) winsorization to replace outliers outlier.pct=0.03, (3) posterior sampling to correct for uneven depth post.sample.no=25, (4) selecting highest *p*-value from output of square-root and fourth-root transformations, (5) reference-based multiple stage normalization (ref.pct=0.5, stage.no=6, excl.pct=0.2), and (6) family-wise error rate control set to true.

### Phage host and lifestyle prediction

The putative host of the phages were predicted using ipHOP^61^ and phage lifestyle was predicted using PhaBox^62^.

### Long-read sequencing

High molecular weight DNA was re-extracted from a fecal sample (40 mg) of LOTUS donor 1 using the Blood and Cell Culture DNA mini kit, genomic-tip 20/G (Qiagen) following the manufacturer’s protocol with minor modifications. Lysis buffer (containing RNase A) was added to the fecal sample and 200 μl of proteinase K was added to the mix. The solution was then vortexed and incubated at 50 °C for 2.5 h (vortexing for 5 secs every 15 min). Following incubation, the mix was loaded onto a Qiagen Genomic-tip and flow through was determined by gravity. The sample was washed three times and eluted with buffer QF. DNA was then precipitated using 0.7 volumes of isopropanol to the eluted DNA. The sample was spun down for 15 min at 4 °C. The DNA pellet was washed with cold 70% ethanol, air-dried and resuspended in molecular grade water overnight. Library preparation was carried out using the ligation sequencing kits (Oxford Nanopore Technologies) SQK-LSK114 for sequencing on the FLO-PRO114M flow cell. Metagenomic sequencing were conducted on a PromethION platform using the software MinKNOW v22.12.5. The generated raw Nanopore data were basecalled using Guppy v6.4.6+ae70e8f.

### Assembly and polishing of long-read sequencing data

Sequencing reads were assembled using Flye 2.9.2-b1786^63^ with the metagenome assembly mode (metaFlye) followed by one round of polishing with medaka v1.8.0 (Oxford Nanopore Technologies). The long-read assemblies were further polished using the short-read polishers Polypolish v0.5.0^64^ and POLCA v4.0.3^65^. To detect their presence in the long-read data, vOTU sequences were then aligned against the non-polished and polished long-read assemblies using BWA-mem v0.7.18 (r1243)^66^.

### vOTU alignment

Alignment of vOTUs of interest from the FOCUS and LOTUS data sets to each other was performed using Clustal Omega^67^. Searches of vOTU fasta sequences was performed using blastn^68^ against the nucleotide collection (nr/nt) limited to taxid: 2 (bacteria) at the National Center for Biotechnology Information. Alignment of vOTUs of interest to the genome of *Oscillospiraceae* CE91-St42 and subsequent visualization was performed using Proksee^69^.

### Construction of metabolic model of *Oscillospiraceae* bacterium CE91-St42

The genome of the bacterium (GenBank: AP025561.1) was annotated in KBase with default parameters using RAST. The model was built using the Build Metabolic Model App implemented in KBase based on the ModelSEED Pipeline for individual genomes^70,71^. The model was gapfilled using the App: MS2 – ‘Improved Gapfill Metabolic Models’. Metabolic output in the form of ModelSEED compound IDs were matched to KEGG IDs (*n* = 747 of 1029 predicted compounds matched) and an enrichment analysis for KEGG pathways was conducted using MBRole 2.0^72^.

### Statistics and Reproducibility

The FOCUS clinical trial was a multicentre, double-blind, randomized, placebo-controlled trial^38^. Patients with active UC were randomly allocated (1:1 ratio) to either FMT or placebo. Patients on placebo were then offered open label FMT. Response was predicted at 60% and 15% for FMT and placebo, respectively, based on limited available data at the time of study design^38^. With an estimated 30% dropouts, *n* = 40 per group was planned to ensure >80% probability of showing a difference between groups, with a two-sided α of 0.05 on modified intention-to-treat analysis^38^. 85 patients were enrolled, of whom 42 and 43 were assigned FMT and placebo, respectively. Of the 85 patients, 81 were treated and 53 with complete sample collection (Tx0, P8 (if applicable), Tx4, Tx8 and TxF) were selected for shotgun metagenomic sequencing. No data were excluded from any analysis.

The LOTUS clinical trial was a double-blind, randomized, placebo-controlled trial^25^. Response was predicted at 36% and 8% for FMT and placebo, respectively, based on previous literature.

With an estimated 15% dropouts, *n* = 32 per group was planned to ensure >80% probability of showing a difference between groups, with a two-sided α of 0.05 on modified intention-to-treat analysis^25^. The study was not powered to detect a difference in the maintenance phase. After 2 weeks of amoxicillin, metronidazole, and doxycycline, patients with active UC were randomly assigned (1:1 ratio) to receive either oral lyophilized FMT (*n* = 15) or placebo (*n* = 20). At week 8, FMT responders were randomly assigned (1:1 ratio) to either continue or withdraw FMT for a further 48 weeks. Recruitment was terminated early due to the COVID-19 pandemic. All available fecal samples from patients were extracted and sequenced, and no data was excluded from any analysis.

### Reporting summary

Further information on research design is available in the Nature Portfolio Reporting Summary linked to this article.

## Supplementary information

Supplementary Information

Reporting Summary

## Source data

Source data

Transparent Peer Review file

## Data Availability

The shotgun metagenomic sequencing data from the FOCUS patients and donors used in this study are available from the European Nucleotide Archive (ENA) under the accession number PRJEB26357. The shotgun metagenomic sequencing data from the LOTUS donors used in this study are available from ENA under the accession number PRJEB50699. The shotgun metagenomic sequencing data from the LOTUS patients (PRJEB58035 [https://www.ebi.ac.uk/ena/browser/view/PRJEB58035]) as well as the long read sequencing data set (PRJEB76864 [https://www.ebi.ac.uk/ena/browser/view/PRJEB76864]) that were generated as part of this study are available from ENA. Processed data in the form of vOTU count tables and classifications for the FOCUS and LOTUS samples are available in Zenodo (accession number 13627782) [10.5281/zenodo.13627782]. Additional metadata is available from the corresponding author. Source data are provided with this paper.

## Code availability

No new code was generated for this study. All software versions and operating procedures have been cited in the Methods.
