# Supplementary material for: The phageome of patients with ulcerative colitis treated with donor fecal microbiota reveals markers associated with disease remission
Source: Nat Commun. 2024 Oct 17;15:8979. doi: 10.1038/s41467-024-53454-4 (PMC11487140; doi:10.1038/s41467-024-53454-4)
Supplement: Supplementary file 1 — Supplementary Information [file 41467_2024_53454_MOESM1_ESM.pdf]

The phageome of patients with ulcerative colitis treated with donor fecal microbiota reveals markers associated with disease remission

Marwan E Majzoub[1#], Sudarshan Paramsothy[2,3#], Craig Haifer[4,5#], Rohit Parthasarathy[1], Thomas J Borody[6], Rupert W Leong[2,3], Michael A Kamm[7,8], Nadeem O Kaakoush[1*]

[1]School of Biomedical Sciences, Faculty of Medicine and Health, UNSW Sydney, NSW 2052, Australia
[2]Concord Clinical School, University of Sydney, Sydney, NSW 2006, Australia
[3]Department of Gastroenterology, Concord Repatriation General Hospital, Sydney, NSW 2139, Australia
[4]School of Clinical Medicine, Faculty of Medicine and Health, UNSW Sydney, NSW 2052, Australia
[5]Department of Gastroenterology, St Vincent's Hospital, Sydney, NSW 2010, Australia
[6]Centre for Digestive Diseases, Sydney, NSW 2046, Australia
[7]Department of Gastroenterology, St Vincent's Hospital, Melbourne, Vic 3065, Australia
[8]Department of Medicine, University of Melbourne, Melbourne, Vic 3010, Australia

[#]These authors contributed equally.

Correspondence:
Scientia A/Prof Nadeem O Kaakoush
School of Biomedical Sciences, Faculty of Medicine and Health
University of New South Wales, Sydney, NSW 2052, Australia
Tel: + 61 2 9065 9728; Email: n.kaakoush@unsw.edu.au

Supplementary Figure 1

Quality assessment of phage identification. A: Jaccard similarity of the donor batches (D1, D5, D6, D10, D11, D14, D15, D17, D19, D20, D22, D24, D25, D28, D29, D31, D32) to the individual donors included (Yes, pink; n=4, 7, 5, 5, 6, 6, 6, 6, 7, 6, 5, 5, 5, 6, 6, 7, 7, respectively) and not included (No, yellow; n=10, 7, 9, 9, 8, 8, 8, 8, 7, 8, 9, 9, 9, 8, 8, 7, 7, respectively) in the batch. Differences were tested using two-way ANOVA with Batch x Inclusion as variables. All FOCUS vOTUs (n=7066) were included in the analysis. Errors are ± SEM. **B:** Pielou's evenness in the individual donors (n=14) and batches (n=17). Differences were tested using a two-sided Welch's t-test (p=0.0009) following assessment of normal distribution with Shapiro-Wilk test (Donors: p=0.6542, Batches: p=0.1368) and variances with F test (p<0.0001). **C:** Principal coordinate analysis (PCO) on Bray-Curtis similarities between individual donor (green) and donor batch samples (purple). Differences between groups were tested using PERMANOVA and PERMDISP. Source data are provided as a Source Data file.

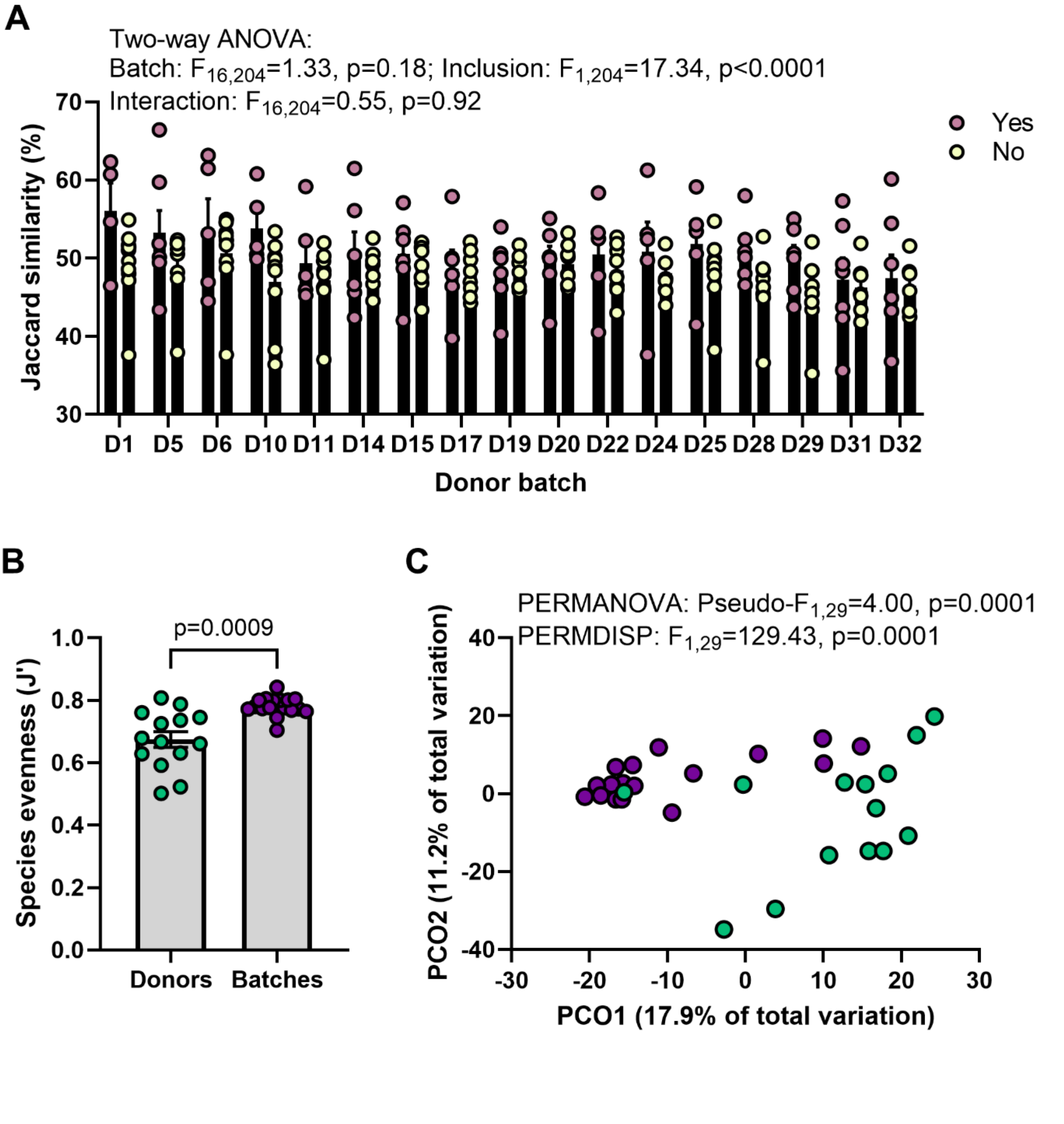

Supplementary Figure 2

Phage diversity in healthy individuals. A: Principal coordinate analysis (PCO) on Bray-Curtis similarities between samples from the individual donors. All FOCUS vOTUs (n=7066) were included in the analysis. 14 donors in FOCUS were included and sampled at their baseline (0 months). 13 of 14 were sampled a second time over a period ranging from 0.3 to 17 months (blue points). **B:** Linear regression between Bray-Curtis similarities intra-donor against time (months). Center line is linear regression equation and broken lines represent 95% confidence intervals. Source data are provided as a Source Data file.

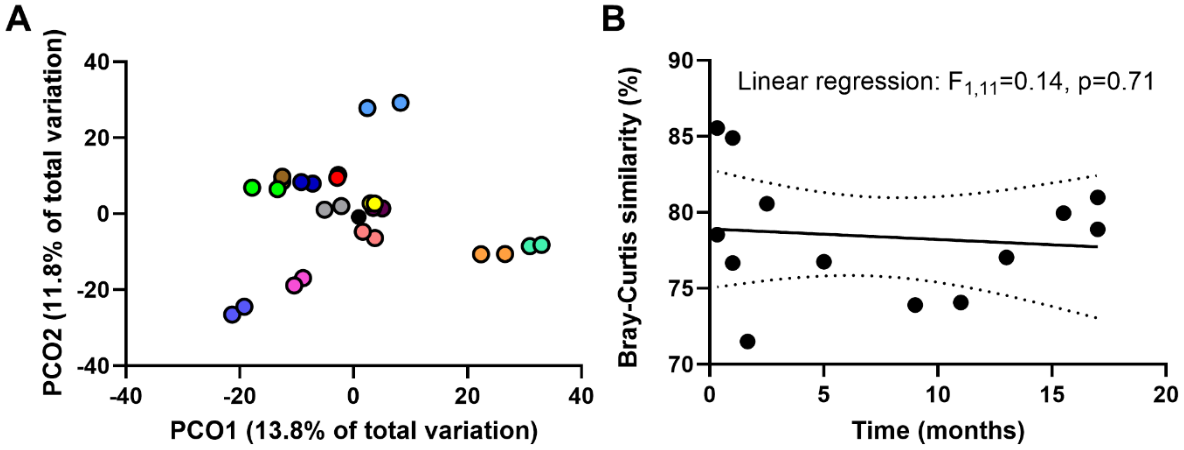

Supplementary Figure 3

Phage beta-diversity in active ulcerative colitis. A: Principal coordinate analysis (PCO) on Bray-Curtis similarities between individual donors (green; n=14) and patient samples (purple; n=53). Differences between groups were tested using PERMANOVA and PERMDISP. FOCUS vOTUs that were classified as a virus (n=2737) were included in the analysis. **B:** Principal coordinate analysis (PCO) on Bray-Curtis similarities between patients at baseline (Baseline, green; n=31) and patients following antibiotic treatment (Post Abx, purple; n=32). Differences between groups were tested using PERMANOVA and PERMDISP. LOTUS vOTUs that were classified as a virus (n=25941) were included in the analysis. Source data are provided as a Source Data file.

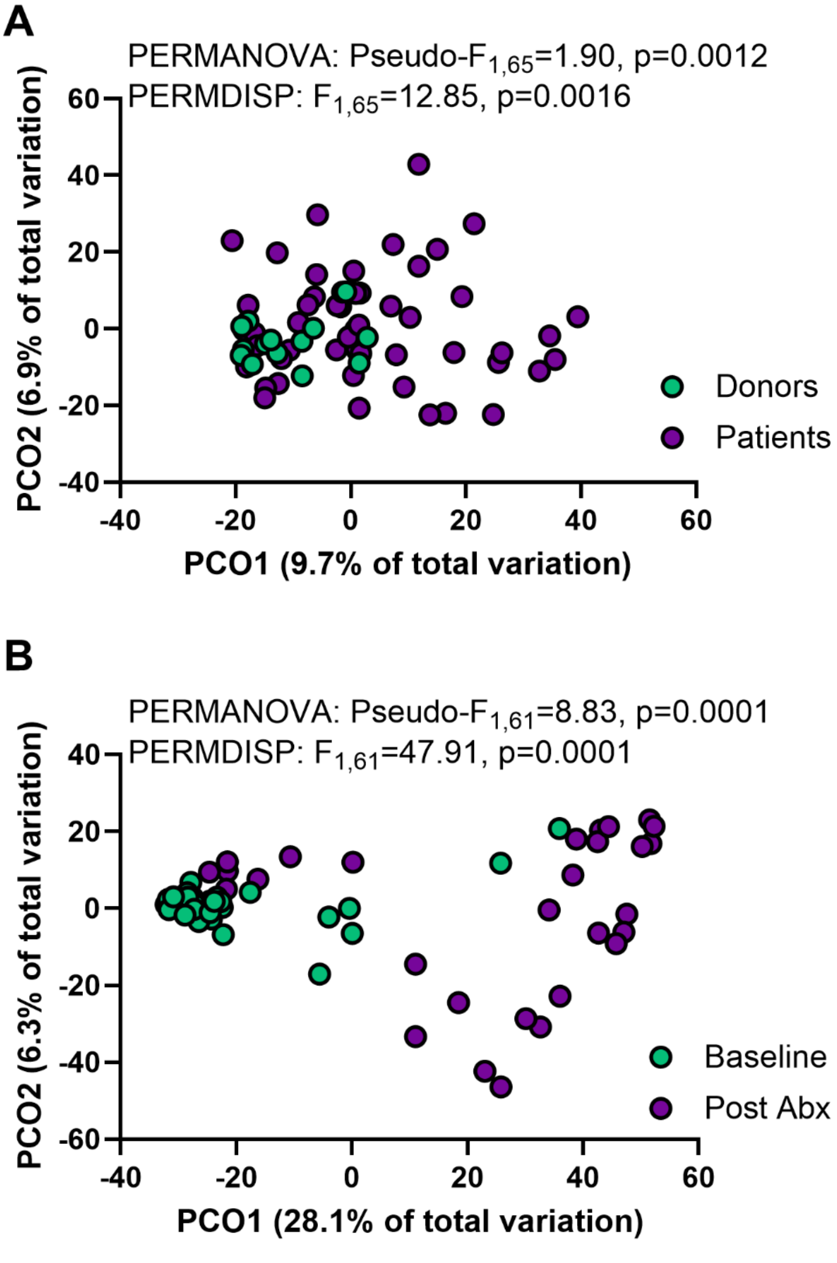

Supplementary Figure 4

Effect of fecal microbiota transplantation on phage diversity. FOCUS and LOTUS vOTUs that were classified as a virus (n=2737 and n=25941, respectively) were included in the analysis where applicable. **A:** Principal coordinate analysis (PCO) on Bray-Curtis similarities for FOCUS samples across all groups. Donor batches (Donors, n=17), patients at baseline (Tx0, n=53), post 8 weeks of placebo (P8, n=21), 4 weeks of FMT (Tx4, n=53), 8 weeks of FMT (Tx8, n=53) and at follow-up 8 weeks following FMT (TxF, n=53). Differences between groups were tested using PERMANOVA and PERMDISP. **B:** Margalef's (species) richness across time during FMT or placebo in LOTUS (including Donors and Baseline). Errors are ± SEM. **C:** Principal coordinate analysis (PCO) on Bray-Curtis similarities for LOTUS samples across all groups. Donors, n=29 samples from 2 donors; Baseline, n=31 patients; Post Abx, n=32 patients; Placebo, n=75 samples; FMT, n=61 samples. Differences between groups were tested using PERMANOVA and PERMDISP. Source data are provided as a Source Data file.

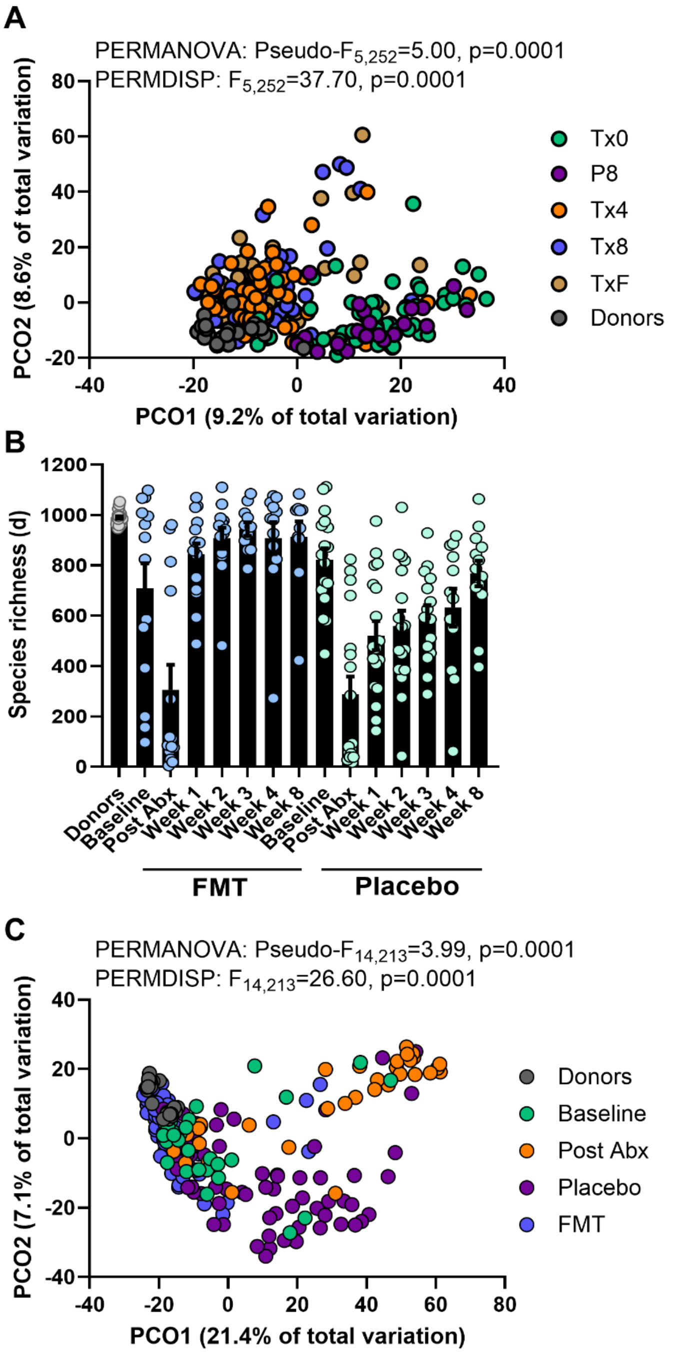

Supplementary Figure 5

Effect of FMT and placebo on relative abundance of vOTUs. FOCUS vOTUs that were classified as a virus (n=2737) were included in the analysis that was performed using ZicoSeq. **A:** Differential abundance analysis between patients at baseline (Tx0; n=53) and patients following 8 weeks of placebo (P8; n=21). **B:** Differential abundance analysis between patients at baseline (Tx0; n=53) and patients following 8 weeks of FMT (Tx8; n=53). **C:** Differential abundance analysis between patients at baseline (Tx0; n=53) and patients at follow-up 8 weeks after FMT treatment (TxF; n=53).

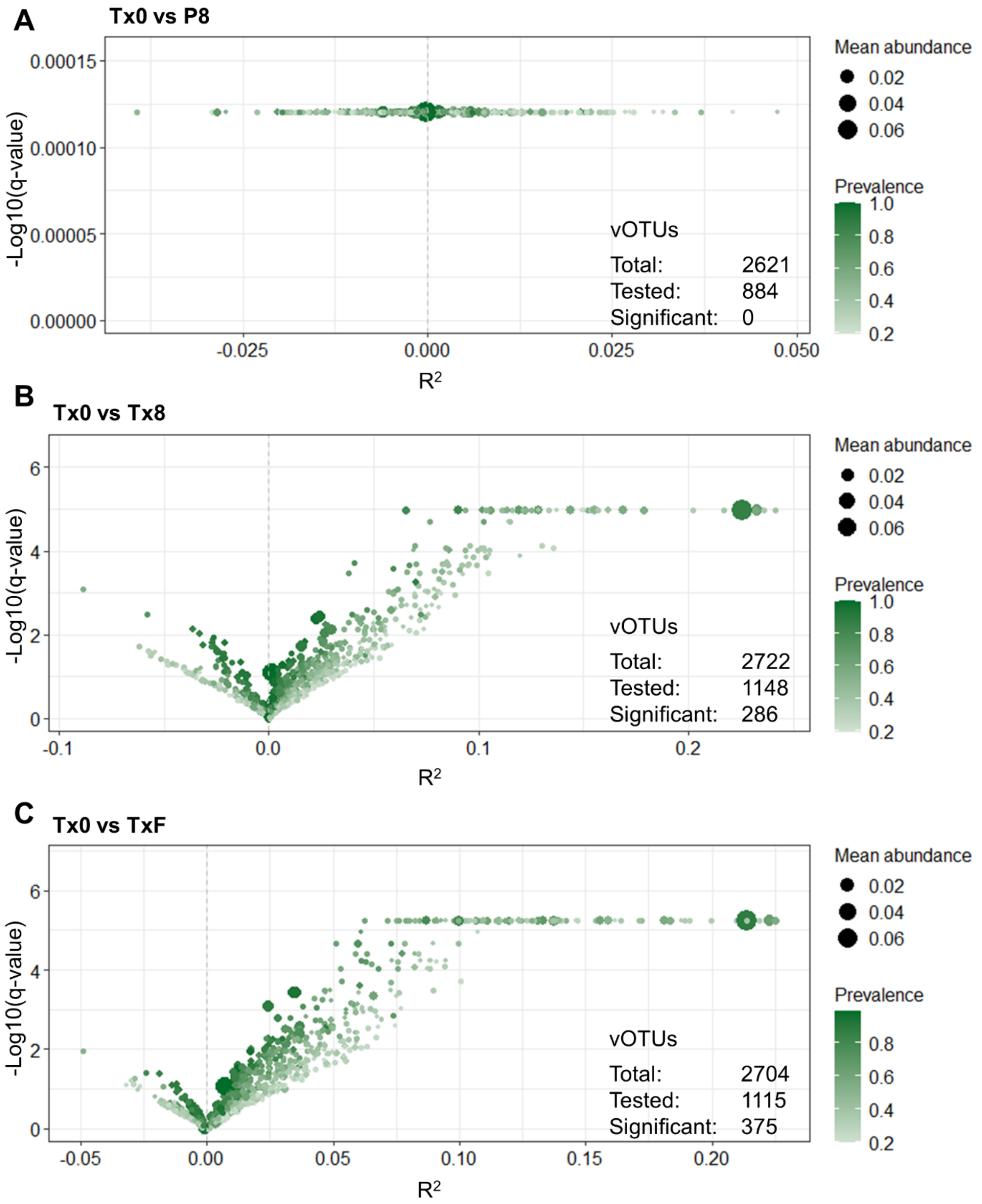

Supplementary Figure 6

Effect of FMT and placebo on relative abundance of vOTUs. LOTUS vOTUs that were classified as a virus (n=25941) were included in the analysis. **A:** Differential abundance analysis between patients at baseline (Baseline; n=17) and patients following 8 weeks of placebo (Post-placebo; n=13). **B:** Differential abundance analysis between patients at baseline (Baseline; n=14) and patients following 8 weeks of FMT (Post-FMT; n=10). **C:** Classification of 46 vOTUs found to be differentially abundant with FMT. Source data are provided as a Source Data file.

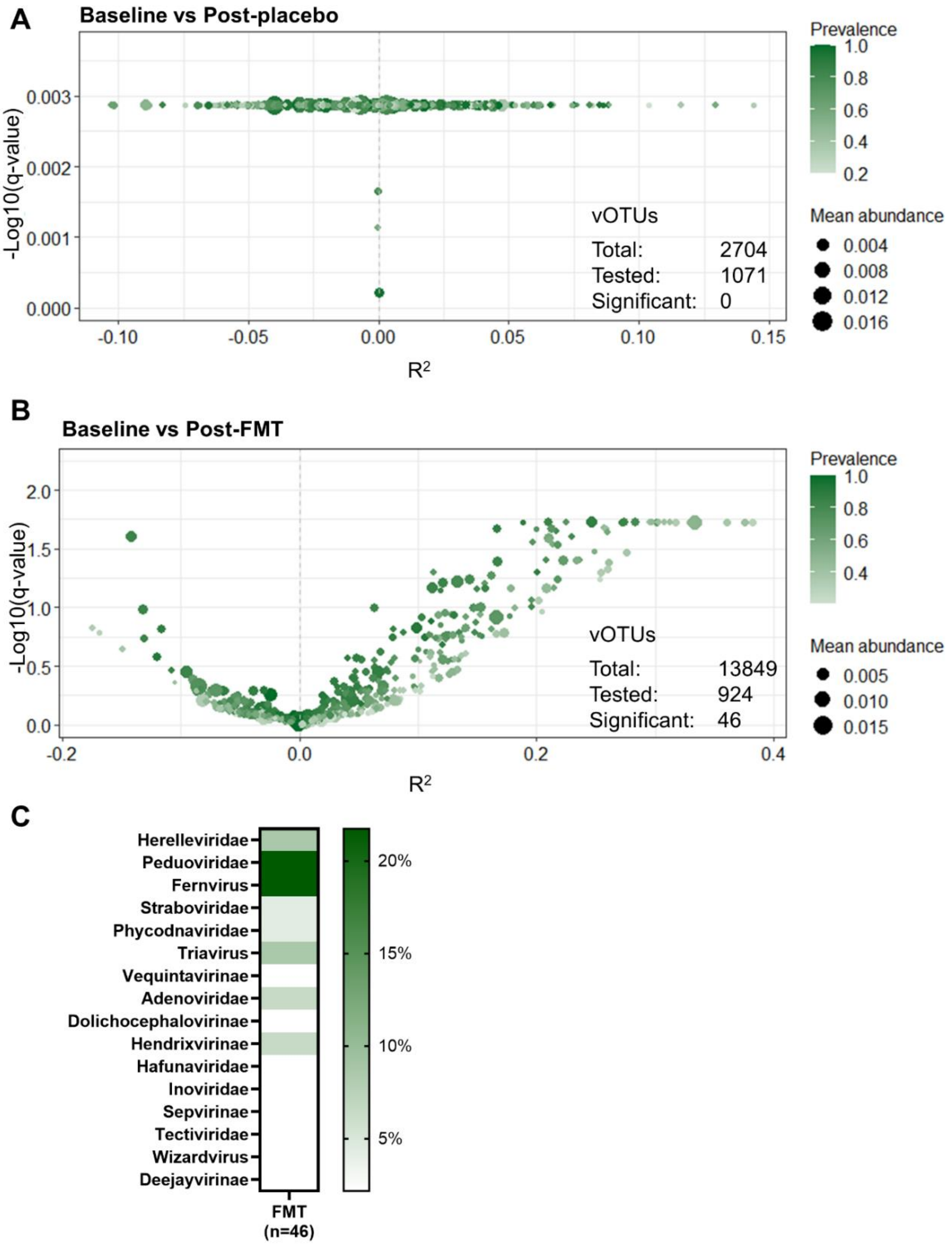

Supplementary Figure 7

Phage diversity and response to fecal microbiota transplantation. FOCUS and LOTUS vOTUs that were classified as a virus (n=2737 and n=25941, respectively) were included in the analysis where applicable. **A:** Principal coordinate analysis (PCO) on Bray-Curtis similarities for FOCUS samples across all groups. Differences between groups were tested using PERMANOVA and PERMDISP. Non-responders at baseline (Tx0N, n=35), responders at baseline (Tx0Y, n=18), non-responders post FMT (Tx8N, n=35) and responders post FMT (Tx8Y, n=18). **B:** Effect of FMT on Margalef's (species) richness in non-responders and responders in the LOTUS trial. Differences were tested with a two-way repeated measures ANOVA and multiple comparisons testing was performed using an uncorrected Fisher's LSD. **C:** Principal coordinate analysis (PCO) on Aitchinson distances between LOTUS donors, responders and non-responders at baseline and post-FMT. Differences between groups were tested using PERMANOVA and PERMDISP. Baseline_n, n=7; Baseline_y, n=7; Post FMT_n, n=7; Post FMT_y, n=7. Source data are provided as a Source Data file.

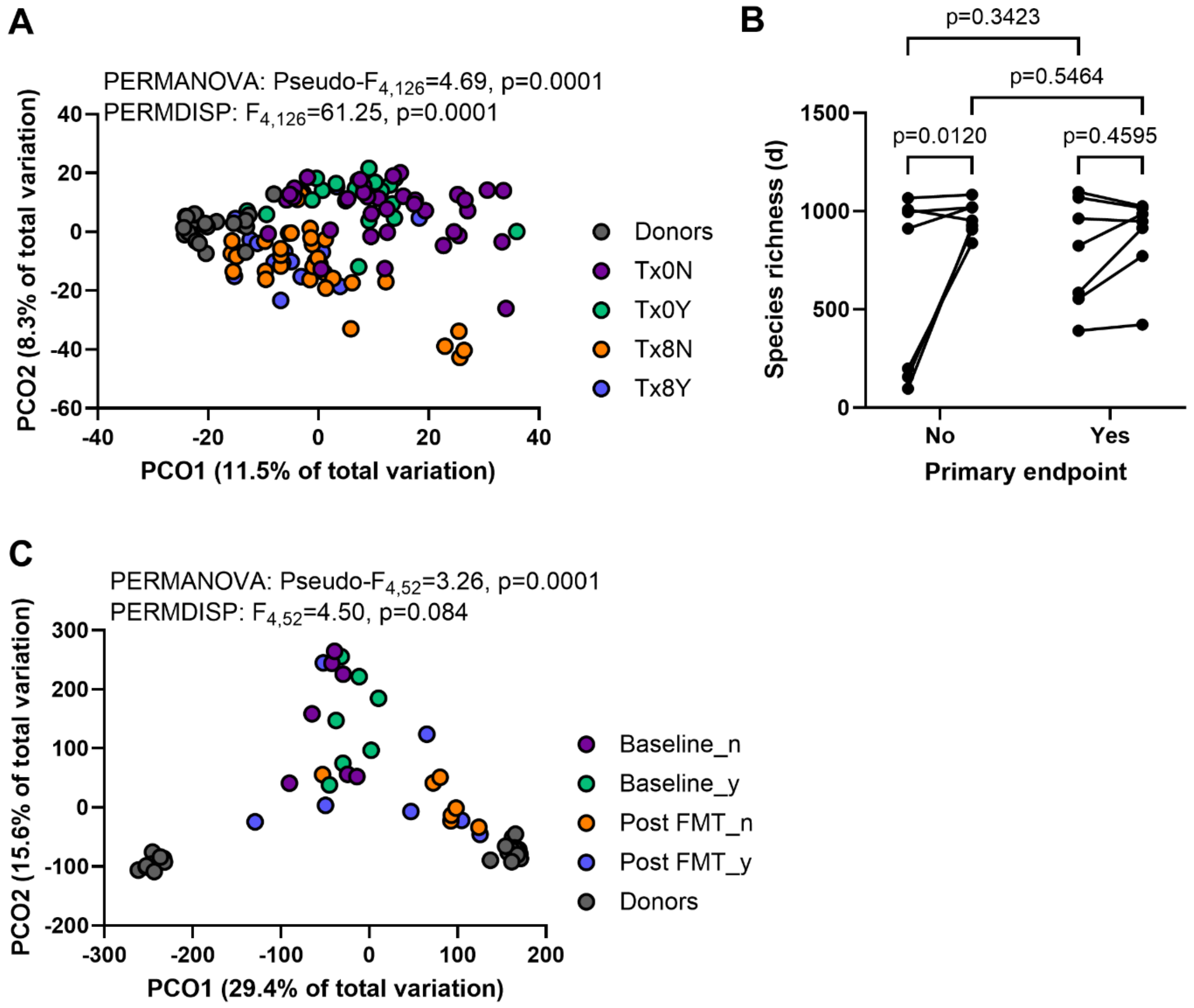

Supplementary Figure 8

Phage diversity during maintenance therapy or withdrawal. LOTUS vOTUs classified as a virus (n=25941) were included in the analysis. **A:** Principal coordinate analysis (PCO) on Bray-Curtis similarities for LOTUS samples across all groups. Differences between groups were tested using PERMANOVA and PERMDISP. FMT_i: patients at end of induction FMT therapy that went into maintenance arm (n=4), FMT_m: patients at end of maintenance FMT therapy (n=4), Flare_i: patients at end of induction FMT therapy that went into withdrawal arm (n=4), Flare_m: patients who withdrew from therapy around the time of disease flare (n=4). **B, C:** Changes in the relative abundances of the remaining LOTUS vOTUs that were homologous to vOTU_151 in FOCUS. Differences were tested with a two-way repeated measures ANOVA and multiple comparisons testing was performed using an uncorrected Fisher's LSD. Source data are provided as a Source Data file.

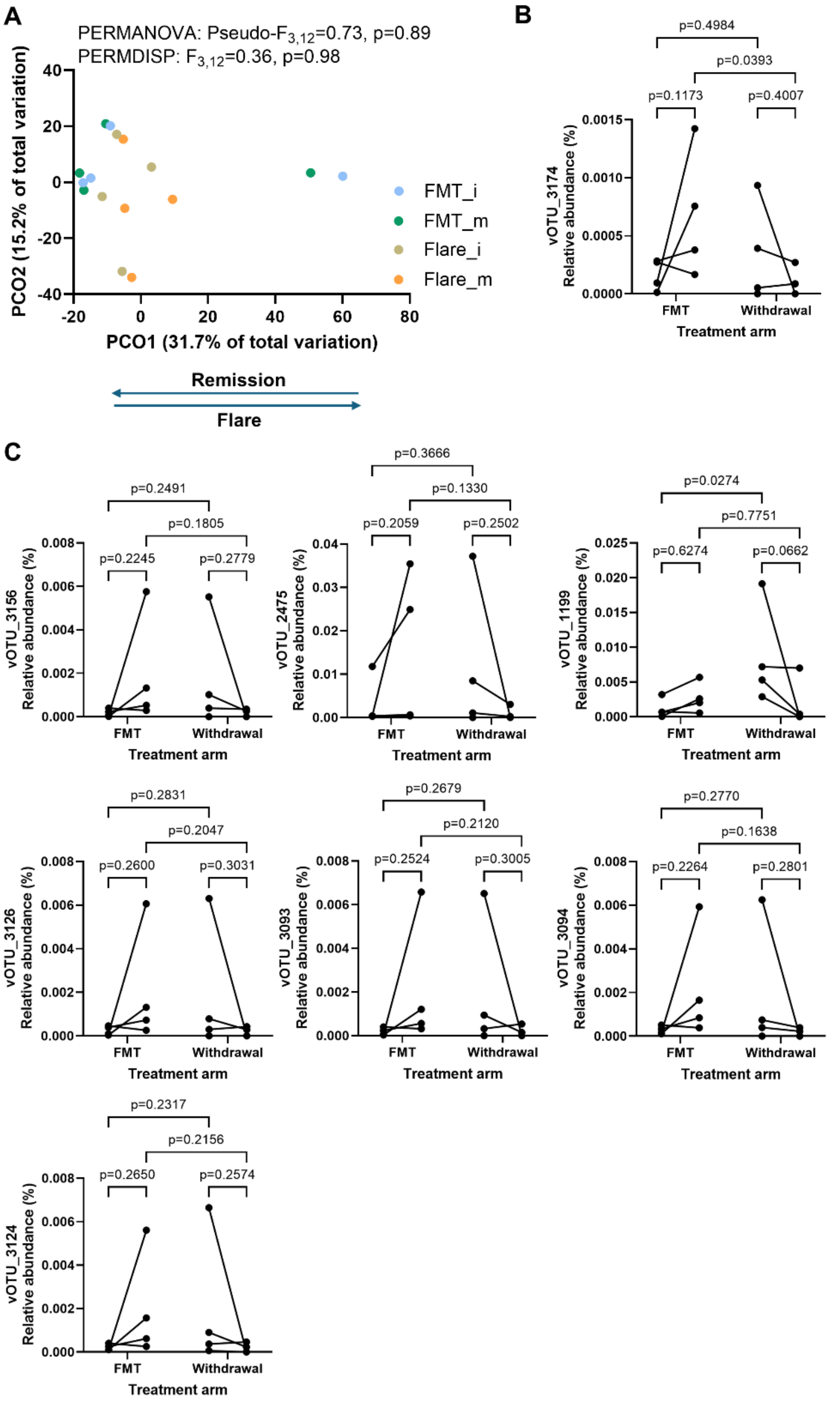

Supplementary Table 1

Results of similarities searches between phages and bacteria. Short-read data: Results of blastn searches between vOTUs of interest from FOCUS and LOTUS and the nucleotide database at NCBI limited to bacterial taxa (taxid: 2). All vOTUs showed high coverage and identity to the assembled genome of isolate *Oscillospiraceae* CE91-St42. Long-read data: Results of blastn searches between long-read contigs of interest and the nucleotide database at NCBI limited to bacterial taxa (taxid: 2). All contigs showed high identity to the assembled genome of isolate *Oscillospiraceae* CE91-St42 but only contig_6261 had high coverage.

**Short-read data**			
	**vOTU ID**	**Blastn hit**	**Coverage / Identity (%)**
	151	*Oscillospiraceae* CE91-St42	98 / 98.7
	3124	*Oscillospiraceae* CE91-St42	83 / 98.68
	2578	*Oscillospiraceae* CE91-St42	89 / 98.71
	3315	*Oscillospiraceae* CE91-St42	94 / 98.32
	3126	*Oscillospiraceae* CE91-St42	99 / 99.61
	3093	*Oscillospiraceae* CE91-St42	99 / 99.5
	3094	*Oscillospiraceae* CE91-St42	99 / 99.37
	2838	*Oscillospiraceae* CE91-St42	99 / 99.52
	3156	*Oscillospiraceae* CE91-St42	99 / 99.5
**Long-read data**			
	**Contig**	**Blastn hit**	**Coverage / Identity (%)**
	6261	*Oscillospiraceae* CE91-St42	99 / 99.37
	7310	*Oscillospiraceae* CE91-St42	24 / 100
	46131	*Oscillospiraceae* CE91-St42	20 / 97.48
	46654	*Oscillospiraceae* CE91-St42	16 / 91.83
	51638	*Oscillospiraceae* CE91-St42	2 / 93.05
