# Supplementary material for: The phageome of patients with ulcerative colitis treated with donor fecal microbiota reveals markers associated with disease remission
Source: Nat Commun. 2024 Oct 17;15:8979. doi: 10.1038/s41467-024-53454-4 (PMC11487140; doi:10.1038/s41467-024-53454-4)
Supplement: Supplementary file 2 — Reporting Summary [file 41467_2024_53454_MOESM2_ESM.pdf]

nature portfolio

Corresponding author(s): Nadeem Kaakoush

Last updated by author(s): Sep 23, 2024

Reporting Summary

Nature Portfolio wishes to improve the reproducibility of the work that we publish. This form provides structure for consistency and transparency in reporting. For further information on Nature Portfolio policies, see our Editorial Policies and the Editorial Policy Checklist.

Statistics

For all statistical analyses, confirm that the following items are present in the figure legend, table legend, main text, or Methods section.

n/a	Confirmed	
☐	☒	The exact sample size (*n*) for each experimental group/condition, given as a discrete number and unit of measurement
☐	☒	A statement on whether measurements were taken from distinct samples or whether the same sample was measured repeatedly
☐	☒	The statistical test(s) used AND whether they are one- or two-sided<br>*Only common tests should be described solely by name; describe more complex techniques in the Methods section.*
☒	☐	A description of all covariates tested
☐	☒	A description of any assumptions or corrections, such as tests of normality and adjustment for multiple comparisons
☐	☒	A full description of the statistical parameters including central tendency (e.g. means) or other basic estimates (e.g. regression coefficient) AND variation (e.g. standard deviation) or associated estimates of uncertainty (e.g. confidence intervals)
☐	☒	For null hypothesis testing, the test statistic (e.g. *F*, *t*, *r*) with confidence intervals, effect sizes, degrees of freedom and *P* value noted<br>*Give P values as exact values whenever suitable.*
☒	☐	For Bayesian analysis, information on the choice of priors and Markov chain Monte Carlo settings
☒	☐	For hierarchical and complex designs, identification of the appropriate level for tests and full reporting of outcomes
☒	☐	Estimates of effect sizes (e.g. Cohen's *d*, Pearson's *r*), indicating how they were calculated

Our web collection on statistics for biologists contains articles on many of the points above.

Software and code

Policy information about availability of computer code

Data collection	Illumina NovaSeq 6000<br>Illumina HiSeq 2500<br>BaseSpace cloud platform<br>Oxford Nanopore Technologies PromethION platform
Data analysis	MetaPhage<br>MEGAHIT v1.2.9<br>metaQuast v5.0.2<br>DeepVirFinder v1.0<br>VIBRANT v1.2.0<br>Phigaro v2.3.0<br>VirSorter2 v2.2.3<br>VirFinder v1.1<br>CD-HIT-EST v4.8.1<br>CheckV v 0.9.0<br>UpSet R library v1.4.0<br>Bowtie2 v2.4.2<br>SAMtools v1.11<br>BamToCov tool v2.0.4

Prodigal v2.6.3
DIAMOND v0.9.14
vConTACT2 v0.9.19
PhaGCN1
PhaGCN2
R v4.3.2
RStudio 2023.12.1 Build 402
Primer-e v6
GraphPad Prism v10
ZicoSeq
PhaBox
ipHOP
MinKNOW v22.12.5
Guppy v6.4.6+ae70e8f
Flye 2.9.2-b1786
medaka v1.8.0
Polypolish v0.5.0
POLCA v4.0.3
BWA-mem v0.7.18 (r1243)
Clustal Omega
Proksee
KBase
MBRole 2.0

For manuscripts utilizing custom algorithms or software that are central to the research but not yet described in published literature, software must be made available to editors and reviewers. We strongly encourage code deposition in a community repository (e.g. GitHub). See the Nature Portfolio guidelines for submitting code & software for further information.

Data

Policy information about availability of data

All manuscripts must include a data availability statement. This statement should provide the following information, where applicable:

- Accession codes, unique identifiers, or web links for publicly available datasets
- A description of any restrictions on data availability
- For clinical datasets or third party data, please ensure that the statement adheres to our policy

The shotgun metagenomic sequencing data from the FOCUS patients and donors used in this study are available from the European Nucleotide Archive (ENA) under the accession number PRJEB26357 [https://www.ebi.ac.uk/ena/browser/view/PRJEB26357]. The shotgun metagenomic sequencing data from the LOTUS donors used in this study are available from ENA under the accession number PRJEB50699 [https://www.ebi.ac.uk/ena/browser/view/PRJEB50699]. The shotgun metagenomic sequencing data from the LOTUS patients (PRJEB58035 [https://www.ebi.ac.uk/ena/browser/view/PRJEB58035]) as well as the long read sequencing data set (PRJEB76864 [https://www.ebi.ac.uk/ena/browser/view/PRJEB76864]) that were generated as part of this study are available from ENA. Processed data in the form of vOTU count tables and classifications for the FOCUS and LOTUS samples are available in Zenodo (accession number 13627782) [DOI: 10.5281/zenodo.13627782]. Additional metadata is available from the corresponding author. Source data are provided with this paper.

Research involving human participants, their data, or biological material

Policy information about studies with human participants or human data. See also policy information about sex, gender (identity/presentation), and sexual orientation and race, ethnicity and racism.

Reporting on sex and gender	Subjects from both male and female sex were recruited to both randomized clinical trials. The microbiome data was not analyzed according to sex as these were double-blind, randomized, clinical trials and not powered to detect sex differences. There are no studies on gender reported in this work.
Reporting on race, ethnicity, or other socially relevant groupings	We did not account for race, ethnicity, or other socially relevant groupings in our analyses.
Population characteristics	FOCUS study:<br>We conducted a multicentre, double-blind, randomized, placebo-controlled trial at three hospitals in Australia. We randomly allocated patients with active ulcerative colitis (Mayo score 4-10) in a 1:1 ratio, using a pre-established randomization list, to either fecal microbiota transplantation or placebo colonoscopic infusion, followed by enemas 5 days per week for 8 weeks. The primary outcome was steroid-free clinical remission with endoscopic remission or response (Mayo score ≤2, all subscores ≤1, and ≥1 point reduction in endoscopy subscore) at week 8. The study protocol is published in Paramsothy, S., et al. Multidonor intensive faecal microbiota transplantation for active ulcerative colitis: a randomised placebo-controlled trial. Lancet 389, 1218-1228 (2017).<br>From November, 2013, to May, 2015, 85 patients were enrolled to our trial, of whom 42 were randomly assigned faecal microbiota transplantation and 43 were allocated placebo. One patient assigned faecal microbiota transplantation and three allocated placebo did not receive study treatment and were excluded from the analysis. Of the 85 patients enrolled, 81 were treated and 53 with complete sample collection were selected for shotgun metagenomic sequencing.<br><br>LOTUS study:<br>We conducted a double-blind, randomized, placebo-controlled trial at two centres in Australia. Eligible patients were aged

	18-75 years with active ulcerative colitis (defined as clinical and endoscopic active ulcerative colitis, with a total Mayo score of 4-10, and a Mayo endoscopic subscore ≥1). After 2 weeks of amoxicillin, metronidazole, and doxycycline, patients were randomly assigned in a 1:1 ratio to receive either oral lyophilised FMT or placebo capsules for 8 weeks, using a pre-specified computer-generated randomization list with a permuted block size of 8. The primary outcome was corticosteroid-free clinical remission with endoscopic remission or response (total Mayo score ≤2, all subscores ≤1, and ≥1 point reduction in endoscopic subscore) at week 8. At week 8, FMT responders were randomly assigned (in a 1:1 ratio, permuted block size of 8) to either continue or withdraw FMT for a further 48 weeks. Analyses were done by modified intention-to-treat, including all patients who received at least one study dose. The study protocol is published in Haifer, C., et al. Lyophilised oral faecal microbiota transplantation for ulcerative colitis (LOTUS): a randomised, double-blind, placebo-controlled trial. Lancet Gastroenterol Hepatol 7: 141-151 (2022).<br>Between May 20, 2019, and March 24, 2020, 35 patients were randomly assigned: 15 to receive FMT and 20 to receive placebo. Recruitment was terminated early due to the COVID-19 pandemic.
Recruitment	FOCUS study:<br>85 patients were recruited to the trial of which 81 were treated. 11 patients were recruited at site 3 where no research samples could be collected. Of the remaining 70 patients, 53 had a complete set of fecal samples collected (baseline, week 4, week 8 and final follow-up), and thus, were selected for shotgun metagenomic sequencing and further analyses.<br><br>LOTUS study:<br>35 patients were recruited to the trial. All available samples from all subjects were sequenced and analyzed in this study.
Ethics oversight	St Vincent's Hospital Sydney Human Research Ethics Committee (both FOCUS and LOTUS)

Note that full information on the approval of the study protocol must also be provided in the manuscript.

Field-specific reporting

Please select the one below that is the best fit for your research. If you are not sure, read the appropriate sections before making your selection.

☒ Life sciences ☐ Behavioural & social sciences ☐ Ecological, evolutionary & environmental sciences

For a reference copy of the document with all sections, see nature.com/documents/nr-reporting-summary-flat.pdf

Life sciences study design

All studies must disclose on these points even when the disclosure is negative.

Sample size	FOCUS study:<br>Based on limited available data and anecdotal experience at the time the study was designed, we predicted remission would be 60% with fecal microbiota transplantation and 15% with placebo; we estimated the proportion of dropouts at 30%. We planned 40 patients per group for recruitment, to ensure a greater than 80% probability of showing a difference between treatment groups, with a two-sided α of 0.05 on modified intention-to-treat analysis. See Paramsothy, S., et al. Multidonor intensive faecal microbiota transplantation for active ulcerative colitis: a randomised placebo-controlled trial. Lancet 389, 1218-1228 (2017) for further information.<br><br>LOTUS study:<br>Our sample size calculation assumed the primary endpoint would be achieved in 36% of patients in the FMT arm versus 8% in the placebo arm, as noted in the FOCUS study. With an estimated dropout rate of 15%, a total of 32 patients per group was planned to ensure an 80% or greater power to demonstrate a difference between treatment groups with a two-sided alpha of 0.05 on modified intention-to-treat analysis. The study was not powered to detect a statistical difference in the exploratory maintenance withdrawal phase of the study. Recruitment was terminated early due to the COVID-19 pandemic. See Haifer, C., et al. Lyophilised oral faecal microbiota transplantation for ulcerative colitis (LOTUS): a randomised, double-blind, placebo-controlled trial. Lancet Gastroenterol Hepatol 7: 141-151 (2022) for further information.
Data exclusions	Patients in the FOCUS study that did not have a complete set of samples (baseline, week 4, week 8 and final follow-up) were excluded from shotgun metagenomic sequencing. All samples that were sequenced were analyzed and none were excluded from downstream statistical analyses.<br><br>All samples collected from patients and donors in the LOTUS study were sequenced and analyzed. None were excluded from downstream statistical analyses.
Replication	The experimental findings were reliably reproduced. See details in figure legends.
Randomization	For both clinical trials, we randomly allocated patients with active ulcerative colitis (Mayo score 4-10) in a 1:1 ratio, using a pre-established randomization list, to either fecal microbiota transplantation or placebo treatment. In the LOTUS study, all patients received antibiotic pre-treatment prior to randomization.
Blinding	Both clinical trials were double-blinded. Patients, treating clinicians, and other study staff were unaware of the assigned treatment. All results presented are based on objective analysis of the capture data, without subjective interpretation.

Reporting for specific materials, systems and methods

We require information from authors about some types of materials, experimental systems and methods used in many studies. Here, indicate whether each material, system or method listed is relevant to your study. If you are not sure if a list item applies to your research, read the appropriate section before selecting a response.

Materials & experimental systems

n/a	Involved in the study
☒	☐ Antibodies
☒	☐ Eukaryotic cell lines
☒	☐ Palaeontology and archaeology
☒	☐ Animals and other organisms
☒	☐ Clinical data
☒	☐ Dual use research of concern
☒	☐ Plants

Methods

n/a	Involved in the study
☒	☐ ChIP-seq
☒	☐ Flow cytometry
☒	☐ MRI-based neuroimaging

Plants

Seed stocks	*Report on the source of all seed stocks or other plant material used. If applicable, state the seed stock centre and catalogue number. If plant specimens were collected from the field, describe the collection location, date and sampling procedures.*
Novel plant genotypes	*Describe the methods by which all novel plant genotypes were produced. This includes those generated by transgenic approaches, gene editing, chemical/radiation-based mutagenesis and hybridization. For transgenic lines, describe the transformation method, the number of independent lines analyzed and the generation upon which experiments were performed. For gene-edited lines, describe the editor used, the endogenous sequence targeted for editing, the targeting guide RNA sequence (if applicable) and how the editor was applied.*
Authentication	*Describe any authentication procedures for each seed stock used or novel genotype generated. Describe any experiments used to assess the effect of a mutation and, where applicable, how potential secondary effects (e.g. second site T-DNA insertions, mosiacism, off-target gene editing) were examined.*
