# Supplementary material for: The phageome of patients with ulcerative colitis treated with donor fecal microbiota reveals markers associated with disease remission
Source: Nat Commun. 2024 Oct 17;15:8979. doi: 10.1038/s41467-024-53454-4 (PMC11487140; doi:10.1038/s41467-024-53454-4)
Supplement: Supplementary file 3 — Source data [file 41467_2024_53454_MOESM3_ESM.pdf]

nature portfolio

Peer Review File

The phageome of patients with ulcerative colitis treated with donor fecal microbiota reveals markers associated with disease remission

Corresponding Author: Professor Nadeem Kaakoush

This file contains all reviewer reports in order by version, followed by all author rebuttals in order by version.

Version 0:

Reviewer comments:

Reviewer #1

(Remarks to the Author)
Brief summary:
In this extensive research article Majzoub et al. looks into finding bacteriophage determinants of responders and non-responders after FMT. One particular temperate phage seems to be associated with successful treatment and are mechanistically supported by a putative B12 production. Only phages are analysed in this study and not the bacteria.
Broad comments and major concerns:
It has been a pleasure reviewing the article, which is well-written, easy to follow, nice figures, and seems proper designed. The story and results are timely and fore sure extremely relevant for the research society. It follows several other studies emphasizing the importance of bacteriophages in health and disease, as well as their therapeutic potential. However, the clinical aspect is outside my expertise. I have a few major concerns that I would like the authors to address, in addition to some minor concerns.
1. My main concern is on the methods of sample preparation of the fecal samples for sequencing. It's not clear for me how the metagenome sequencing differentiate temperate phages from prophages. If the samples had undergone virome separation, where free viruses and bacteria are separated (https://www.mdpi.com/1999-4915/11/7/667) and thereafter treated with DNase to remove free nucleotides, then you will have generated a metavirome reflecting only the free viral particles. How does the authors' wet- and dry-lab approach account for this? Maybe I just miss something, but I cannot understand how. The method used could potentially have impact on how the results should be interpreted.
2. In figure 4D: Its important in the text to emphasize that the vOTUs are not only representing bacteriophages, but also some of the families listed here are eukaryotic viruses like Parvoviridae, Adenoviridae, Circoviridae etc, and maybe other that I have missed, e.g. there is a clear change in Adenoviridae that should be addressed and also included in the discussion. Without having checked all of them, archaeal viruses could also be among the list.
3. I believe that the finding of one phage with less 0.003% relative abundance being a determinant of a successful response on FMT, should be toned down. It may be important, and maybe there are many more that together could be a strong indicator, but for now this finding is limited, although very important and worth publishing. I do acknowledge that the B12 part of the story indeed support the claim of the phage being important, and this is also why your finding is really interesting.
4. Line 345-350: Do you have B12 levels from the recipients to compare with other studies, and whether this support your phage-B12 hypothesis? If you have the data it should be included, and if not, state that you do not have it as a limitation of the study.
5.
Minor concerns
1. Why are the gut bacteria not mentioned in the article? Is this due to the FOCUS and LOTUS being published already? I might have missed that, so just ensure that it have been stated.
2. In figure 3C: It could be argued whether its biological meaningful to include vOTUs with less than 2 log(2) fold differences, despite it being significant. Especially vOTU0465 and 0114.
3. Line 237: Low dose FMT – define "low". Is that based on gram, volume, CFU count, or FMT frequency?
4. Fig 7C. Kinda similar comment here as Fig. 3C. Relative abundance of a phage being below 0.003%, is hard to say whether this is biological relevant or not – maybe it is, but considering the low abundance, I would suggest the authors tone down a bit the importance of this observation. It should surely be included and discussed, but it could also be a by-chance finding – also considering that usually vOTUs tables consists of 5000+ contigs, which is impossible to perform post hoc tests

on.
5. In the table of Fig 8B. Add “Predicted host” instead of just “host”, since you have used Iphop in the prediction.
6. Line 322: Speculate of why your study reported an effect on phage richness and composition, while others didn’t.
7. Line 356-358: The questions could also be whether it should be the bacterial species including the prophages or just the temperate phage alone that could be used for supplementation?
8. The sample input (in mg) for the Illumina short-read sequencing should be stated.
9. I miss a more daring and bold hypothesis/speculation in the discussion of what your results potentially could lead to and be used for in other clinical studies. E.g. could phage profiles be a part of a standard screening process and not just safety? And in relation to the Ott et al. 2017 that you cite in the introduction, whether donor filtrates could be used instead of FMT? Especially considering the story of this article is only focusing on the phages and not the bacteria.

Reviewer #2

(Remarks to the Author)
This is an interesting, largely a descriptive paper of the changes that are observed in the phageome following FMT, or antiobiotic treatment.

Some critical information that will help the reader assess the validity of the findings is missing. Specifically, there is no information about the metagenomic sequencing depth that was used to generate the underlying datasets in this study. The importance of this is reinforced when the authors state “Line 117: “The variation in vOTU detection was likely a consequence of LOTUS samples being sequenced at a depth substantially greater than FOCUS samples, and this was a key reason why the data sets were examined independently”. The lack of information on the ranges of read depths affects the assessment and reproducibility of the work.

Moreover, in the early stages of the manuscript (Line 105) the authors state “FOCUS trial, patients with UC at baseline (Tx0), week 4 FMT (Tx4), week 8 FMT (Tx8) and final follow-up 8 weeks following FMT (TxF) as well as their donors (both donor batches and the individual donors contributing to the batches) were profiled” and in the methods “samples include patients at baseline (Tx0, n=53), patients at week 4 FMT (Tx4, 366 n=53), patients at week 8 FMT (Tx8, n=53), patients 8 weeks following FMT (TxF, n=53)”. It would help the reader if the authors could simplify this and clarify the relationships between these sample time points especially “patients 8 weeks following FMT” and “patients at week 8”.

Figure 2 A – sampling profile is difficult to understand. There is no information to identify repeated sampling of the same donor. The top figure only has 13 points yet n=14.

Figure 2C – 4 points fall well outside of what I assume is the 95% CI (broken lines – not annotated in the figure or legend) – the authors do not talk about these.

In the discussion the authors state “We identify that bacteriophage populations are stable in health, dysbiotic in disease,… “ This statement is misleading as it implies that the phageome population is not stable in the UC disease state. Differences from healthy do not mean the diseased state phageome is not stable. This was not measured in this study and should be clarified.

The strength of this analysis is that these donors are validated as healthy through an extensive testing regimen 27, which is a step beyond standard healthy controls recruited to case-control studies. There are several case-control studies have done just that. Moreover, a quick google search identified that at least one has performed a similar analysis to this study; Zuppi et al. Fecal microbiota transplantation alters gut phage communities in a clinical trial for obesity. Microbiome 12, 122 (2024).

Discussion of Oscillospiraceae strain is very speculative and might be better considered putative until there is empirical evidence of causality. Did the authors identify Oscillospiraceae within the microbial metagenomic analysis (before the phage analysis) that linked it to the clinical resolution? Again, discussion of this, bearing in mind the sequencing depth of these studies, would be helpful for the reader.

The methods are clear and able to be followed.

Project PRJEB26357 consists of 284 files metagenome. However, I was unable to find any metadata. The LOTUS data is unavailable, and the project number has not been provided. These two points make it impossible for anyone to repeat this work. Additionally, there is no statement that the authors agree to make this information available upon request.

Version 1:

Reviewer comments:

Reviewer #1

(Remarks to the Author)
I would thank the authors for properly addressing the concerns of both my review and Reviewer #2.
I'm overall satisfied with the responses. I just want to add the below minor comments. You only need to address Q3. In the

below I'll refer to the different comments as my initially major concerns Q1-Q4.

Q1: Thanks for addressing this. As I read the the procotol from the supplier it appear as sufficient for the purpose.

Q2: It is quite normal finding these eukaryotic viruses in fecal matter, so what you have done now is sufficient. It just have to be addressed.

Q3: Okay, I see its a fine argument in regard of the genetic overlap between the different vOTUs that likely are from the same phage. However, it would require long-read sequencing to do that like Nanopore sequencing, and in term of a more quantatively measurement, I would recommend you considering follow this paper: https://microbiomejournal.biomedcentral.com/articles/10.1186/s40168-018-0446-z - which is something you could add to the limitations as a future solution.

Q4: Okay, fine with the limitations.

I wish you all the best luck in your future research.

Reviewer #2

(Remarks to the Author)
I would like to thank the authors for their responses. The additional information they provided has improved the manuscript.

Open Access This Peer Review File is licensed under a Creative Commons Attribution 4.0 International License, which permits use, sharing, adaptation, distribution and reproduction in any medium or format, as long as you give appropriate credit to the original author(s) and the source, provide a link to the Creative Commons license, and indicate if changes were made.
In cases where reviewers are anonymous, credit should be given to 'Anonymous Referee' and the source.
The images or other third party material in this Peer Review File are included in the article's Creative Commons license, unless indicated otherwise in a credit line to the material. If material is not included in the article's Creative Commons license and your intended use is not permitted by statutory regulation or exceeds the permitted use, you will need to obtain permission directly from the copyright holder.
To view a copy of this license, visit https://creativecommons.org/licenses/by/4.0/

We thank the editors and reviewers for the constructive comments on our manuscript and the opportunity to submit a revised version for consideration.

Reviewer #1:

Brief summary:
In this extensive research article Majzoub et al. looks into finding bacteriophage determinants of responders and non-responders after FMT. One particular temperate phage seems to be associated with successful treatment and are mechanistically supported by a putative B12 production. Only phages are analysed in this study and not the bacteria.

Broad comments and major concerns:
It has been a pleasure reviewing the article, which is well-written, easy to follow, nice figures, and seems proper designed. The story and results are timely and fore sure extremely relevant for the research society. It follows several other studies emphasizing the importance of bacteriophages in health and disease, as well as their therapeutic potential. However, the clinical aspect is outside my expertise. I have a few major concerns that I would like the authors to address, in addition to some minor concerns.

Thank you for the kind words on our manuscript and the constructive review. We hope that we have addressed all your concerns below.

1. My main concern is on the methods of sample preparation of the fecal samples for sequencing. It's not clear for me how the metagenome sequencing differentiate temperate phages from prophages. If the samples had undergone virome separation, where free viruses and bacteria are separated (https://www.mdpi.com/1999-4915/11/7/667) and thereafter treated with DNase to remove free nucleotides, then you will have generated a metavirome reflecting only the free viral particles. How does the authors' wet- and dry-lab approach account for this? Maybe I just miss something, but I cannot understand how. The method used could potentially have impact on how the results should be interpreted.

Our analysis was conducted on shotgun metagenomic sequencing data from total DNA extracts (i.e., without prior virus-like particle purification). We agree that this is an important point to clarify, and that the method does not allow for the differentiation between temperate phages and prophages. We have revised text in the Introduction, Methods and Discussion to this end. The new text reads:

Introduction:
"Thus, here, we analyzed the phageome of patients and donors recruited to two independent randomized clinical trials of FMT in UC using shotgun metagenomics on fecal DNA extracts without prior virus-like particle (VLP) purification."

Discussion:
"The study has several limitations. Our findings are from shotgun metagenomic sequencing with no prior VLP purification, meaning the data cannot differentiate between temperate phages and prophages."

Methods:
"At the time, total DNA was extracted from fecal samples using the MOBIO PowerViral RNA/DNA Isolation kit (Catalog no. 28000-BUNDLE) without prior VLP purification."

"Total DNA was extracted from the fecal samples using the QIAamp PowerFecal DNA Kit (Qiagen; Chadstone, Vic, AU). Sequencing of total DNA (no prior VLP purification) was performed at the Ramaciotti Centre for Genomics (UNSW Sydney)…"

2. In figure 4D: Its important in the text to emphasize that the vOTUs are not only representing bacteriophages, but also some of the families listed here are eukaryotic viruses like Parvoviridae, Adenoviridae, Circoviridae etc, and maybe other that I have missed, e.g. there is a clear change in Adenoviridae that should be addressed and also included in the discussion. Without having checked all of them, archaeal viruses could also be among the list.

Thank you for highlighting this. We have now included a sentence in the Results to address this. We have also added text to the Discussion & Limitations to expand on this point. The new text reads:

Results:
"Of note, several vOTUs found to be differentially abundant following antibiotic treatment were classified to eukaryotic DNA viral families including *Adenoviridae*, *Circoviridae* and *Parvoviridae* (Figure 4D)."

Discussion:
"Among the vOTUs that were found to be differentially abundant with antibiotics were several that were classified to eukaryotic DNA viruses such as *Adenoviridae*, *Circoviridae* and *Parvoviridae*. The importance of this finding remains unclear but the presence of these viruses at baseline in patients with active UC should be explored further."

Limitations:
"As a result of sequencing DNA, we attempted to limit our viral analysis to phages but several eukaryotic DNA viruses were also identified."

3. I believe that the finding of one phage with less 0.003% relative abundance being a determinant of a successful response on FMT, should be toned down. It may be important, and maybe there are many more that together could be a strong indicator, but for now this finding is limited, although very important and worth publishing. I do acknowledge that the B12 part of the story indeed support the claim of the phage being important, and this is also why your finding is really interesting.

The average relative abundance of vOTU_151 across all FOCUS samples (n=285) even when including non-classified vOTUs is 0.0233%. In the LOTUS samples, the depth of sequencing resulted in the same phage being split into several vOTUs (please see Figure 8A). We believe this is the cause of the low relative abundance per vOTU and the true relative abundance of the phage is the sum of relative abundances of the relevant vOTUs. This amounts to similar levels as those observed in the FOCUS samples. However, we do agree with the reviewer that the findings still need to be toned down due to the relative abundance of the phage. We have gone through the manuscript and included text modifications to the Abstract, Introduction and Discussion to this end as requested. The modified text reads:

Abstract:
"We identify a donor bacteriophage putatively associated with disease remission, which on genomic analysis was found integrated in a bacterium classified to

***Oscillospiraceae*, previously isolated from a centenarian and predicted to produce vitamin B complex except B12.”**

Introduction:
“We were also able to determine the impact of antibiotic treatment and FMT on bacteriophage diversity and composition, as well as identify putative novel determinants of disease remission.”

Discussion:
“We found specific bacteriophages that were putatively associated with disease remission, and on further analysis, they represented a phage detected in the genome of a bacterial taxon classified to *Oscillospiraceae* and previously isolated from a Japanese centenarian[26].”

4. Line 345-350: Do you have B12 levels from the recipients to compare with other studies, and whether this support your phage-B12 hypothesis? If you have the data it should be included, and if not, state that you do not have it as a limitation of the study.

Unfortunately we do not have B12 levels from our patients. We have included this as a limitation of the study as requested. The new text reads:

“We could not confirm the importance of levels of vitamin B complex in our patients as these measurements were not available. Future work should explore the relationship between this phage and levels of vitamin B complex.”

Minor concerns
1. Why are the gut bacteria not mentioned in the article? Is this due to the FOCUS and LOTUS being published already? I might have missed that, so just ensure that it have been stated.

As there is a significant body of literature exploring the bacterial component of the gut microbiota and its role in FMT in UC, we wanted to focus on the phage component as there are not many studies assessing the role of phages in this context. We have also previously assessed gut bacterial changes in the FOCUS patients and donors as well as the LOTUS donors. This information has been included in the revised Methods. The added text reads:

FOCUS:
“The bacterial component of the gut microbiome in patients and donors was assessed previously.[38]”

LOTUS:
“The bacterial component of the gut microbiome in the donors was assessed previously.[39]”

2. In figure 3C: It could be argued whether its biological meaningful to include vOTUs with less than 2 log(2) fold differences, despite it being significant. Especially vOTU0465 and 0114.

Thank you for this point. We did not want to implement any additional filtration steps based on Log2FC to present all significant findings as is. However, to account for the

reviewer's concern, we have included a statement to address this in the revised manuscript. The added text reads:

"While statistically significant, caution should be taken in the interpretation of vOTUs with <2 Log2FC (n=14), particularly vOTU465 and vOTU114."

3. Line 237: Low dose FMT – define "low". Is that based on gram, volume, CFU count, or FMT frequency?

Low dose is defined by the number of capsules the patient received daily in the maintenance phase relative to what they received in the induction phase. In the maintenance phase, patients received 2 capsules daily. This has been included into the revised manuscript with a reference to the clinical trial article that includes the above information. The added text reads:

"Low dose maintenance FMT (2 capsules daily) was defined relative to the number of capsules patients received in the induction phase.[25]"

4. Fig 7C. Kinda similar comment here as Fig. 3C. Relative abundance of a phage being below 0.003%, is hard to say whether this is biological relevant or not – maybe it is, but considering the low abundance, I would suggest the authors tone down a bit the importance of this observation. It should surely be included and discussed, but it could also be a by-chance finding – also considering that usually vOTUs tables consists of 5000+ contigs, which is impossible to perform post hoc tests on.

We have adjusted the conclusion of this section to tone down the importance of this finding as requested. The modified and added text reads:

"However, caution should be taken when interpreting these findings as the relative abundances of the vOTUs are low and multiple comparison corrections have not been performed."

5. In the table of Fig 8B. Add "Predicted host" instead of just "host", since you have used Iphop in the prediction.

We have replaced "Host" with "Predicted host" in the revised Figure 8B as requested.

6. Line 322: Speculate of why your study reported an effect on phage richness and composition, while others didn't.

We have now included further discussion on why we believe this is the case. The new text reads:

"We speculate that in the FOCUS study this may be due to the transplant of supraphysiological levels of phages that exist within the donor batches (made up of several individual donors), whereas in the LOTUS study, this was the result of prior antibiotic depletion of phage population richness."

7. Line 356-358: The questions could also be whether it should be the bacterial species including the prophages or just the temperate phage alone that could be used for supplementation?

This is an important point, thank you. We have now adjusted our conclusion to highlight this. The revised text reads:

"Establishing the potential therapeutic benefits of supplementation with the bacterial species corresponding to *Oscillospiraceae* bacterium CE91-St42, or the temperate phage integrated within the bacterium, in patients with UC may prove to be an exciting outcome of this work."

8. The sample input (in mg) for the Illumina short-read sequencing should be stated.

DNA input was according to the specifications of the Illumina kits used at the time (Nextera XT for FOCUS and Illumina DNA prep for LOTUS). We have now included this information in the Methods section of the revised manuscript. The new text reads:

"Patients and donor samples from the FOCUS clinical trial (n=285) were previously profiled using shotgun metagenomic sequencing on an Illumina HiSeq 2500 with 2x250bp chemistry (DNA input of 1 ng) at the Ramaciotti Centre for Genomics[37,38]."

"Sequencing was performed at the Ramaciotti Centre for Genomics (UNSW Sydney) where DNA was prepared using Illumina DNA prep kits (Illumina; Melbourne, Vic, AU) and sequenced on an Illumina NovaSeq 6000 S4 run using 2x150bp chemistry (DNA input of 500 ng)."

9. I miss a more daring and bold hypothesis/speculation in the discussion of what your results potentially could lead to and be used for in other clinical studies. E.g. could phage profiles be a part of a standard screening process and not just safety? And in relation to the Ott et al. 2017 that you cite in the introduction, whether donor filtrates could be used instead of FMT? Especially considering the story of this article is only focusing on the phages and not the bacteria.

We have now included some of the points the reviewer has mentioned in the revised Discussion section. The revised and new text read:

"This highlighted the importance of phage populations in the context of UC and microbiome manipulation as well as the ability of phage profiling to putatively identify novel bacterial taxa that could be determinants of response to FMT."

"Future work could also consider the utility of these phage biomarkers for the selection of optimal FMT donors as well as testing the ability of the sterile stool filtrates from these donors to induce remission in patients with UC."

Reviewer #2:

This is an interesting, largely a descriptive paper of the changes that are observed in the phageome following FMT, or antiobiotic treatment.

Thank you for your interest in our manuscript and the constructive review. We hope that we have addressed all your concerns below.

Some critical information that will help the reader assess the validity of the findings is missing. Specifically, there is no information about the metagenomic sequencing depth that was used to generate the underlying datasets in this study. The importance of this is reinforced when the authors state "Line 117: "The variation in vOTU detection was likely a consequence of LOTUS samples being sequenced at a depth substantially greater than FOCUS samples, and this was a key reason why the data sets were examined independently". The lack of information on the ranges of read depths affects the assessment and reproducibility of the work.

We have now included the average read depth per data set into the manuscript as requested. The added text now reads:

"The variation in vOTU detection was likely a consequence of LOTUS samples being sequenced at a depth substantially greater than FOCUS samples (average read depth ± SEM: FOCUS: 3415827 ± 37684; LOTUS: 36799073 ± 1186931), and this was a key reason why the data sets were examined independently."

Moreover, in the early stages of the manuscript (Line 105) the authors state "FOCUS trial, patients with UC at baseline (Tx0), week 4 FMT (Tx4), week 8 FMT (Tx8) and final follow-up 8 weeks following FMT (TxF) as well as their donors (both donor batches and the individual donors contributing to the batches) were profiled" and in the methods "samples include patients at baseline (Tx0, n=53), patients at week 4 FMT (Tx4, 366 n=53), patients at week 8 FMT (Tx8, n=53), patients 8 weeks following FMT (TxF, n=53)". It would help the reader if the authors could simplify this and clarify the relationships between these sample time points especially "patients 8 weeks following FMT" and "patients at week 8".

We have attempted to clarify the relationships between the sample time points as requested. The first statement was modified to:

"In the first data set from the FOCUS trial, patients with UC at baseline (Tx0), week 4 of FMT (Tx4), week 8 of FMT (Tx8) and final follow-up 8 weeks after FMT treatment had concluded (TxF) as well as their donors (both donor batches and the individual donors contributing to the batches) were profiled."

The second statement was modified to:

"When combining the blinded and open-label arms of the trial, samples include patients at baseline (Tx0, n=53), the same patients at week 4 of FMT (Tx4, n=53), the same patients at week 8 of FMT (Tx8, n=53), the same patients 8 weeks after the conclusion of FMT treatment (TxF, n=53), individual donors recruited to the trial (27 samples from n=14 donors), and the multi-donor batches transplanted into the patients (25 samples from n=17 batches)."

Figure 2 A – sampling profile is difficult to understand. There is no information to identify repeated sampling of the same donor. The top figure only has 13 points yet n=14.

We had initially stated in the legend of Figure 2A that 13 of the 14 donors were sampled twice, hence only 13 points despite there being 14 donors. However, we agree that the figure legend can be improved. The revised legend reads:

"Sampling from the donors in the FOCUS trial (top) and LOTUS trial (bottom). 14 donors in FOCUS were included and sampled at their baseline (0 months). 13 of 14 were sampled a second time over a period ranging from 0.3 to 17 months (blue points). Two donors in LOTUS were included that were sampled over 44 weeks (donor 1, n=12, pink) and 70 weeks (donor 2, n=17, blue)."

Figure 2C – 4 points fall well outside of what I assume is the 95% CI (broken lines – not annotated in the figure or legend) – the authors do not talk about these.

Thank you for pointing this out. We have now annotated the broken lines in the legend of Figure 2C. We have also included additional text referring to the four points that fall outside the 95% CI. The new text reads:

"While most repeat samples from the donors showed 70-80% similarity in phage composition intra-donor over time, it is worth noting that two donors showed higher than average similarity (>80%) whereas another two fell below average (<70%) (Figure 2C)."

In the discussion the authors state "We identify that bacteriophage populations are stable in health, dysbiotic in disease,… " This statement is misleading as it implies that the phageome population is not stable in the UC disease state. Differences from healthy do not mean the diseased state phageome is not stable. This was not measured in this study and should be clarified.

We have now adjusted the statement to address this concern. The revised statement reads:

"We identify that bacteriophage populations are stable in healthy individuals, altered in richness and composition in patients with active UC, can be modulated with antibiotics and FMT in these patients, as has been reported for the bacterial component of the microbiome."

The strength of this analysis is that these donors are validated as healthy through an extensive testing regimen 27, which is a step beyond standard healthy controls recruited to case-control studies. There are several case-control studies have done just that. Moreover, a quick google search identified that at least one has performed a similar analysis to this study; Zuppi et al. Fecal microbiota transplantation alters gut phage communities in a clinical trial for obesity. Microbiome 12, 122 (2024).

Apologies, we are unfamiliar with any case-control study assessing phages that has required their recruited controls to undergo the extensive baseline and subsequent monthly testing that FMT donors undergo to be approved to donate. We would imagine that a very valid reason such as FMT recipient safety is required to subject healthy individuals to such a rigorous testing regimen. Nevertheless, we have revised the statement to alleviate this concern. The revised text removes any mention of case-control studies and reads:

“The strength of this analysis is that these donors are validated as healthy through an extensive testing regimen[27].”

Regarding Zuppi *et al*, this work was made available online on July 6th approximately three weeks after the submission of our manuscript. We have now cited this work in our Discussion. The new text reads:

“Our findings are also in line with a recent study by Zuppi *et al* that explored the impact of FMT on the phageome of recipients being treated for obesity and comorbidities[30]. The authors reported donor phage engraftment leading to a shift in recipient phageome composition towards the donors and an increase in phage richness in females following FMT[30].”

Discussion of Oscillospiraceae strain is very speculative and might be better considered putative until there is empirical evidence of causality. Did the authors identify Oscillospiraceae within the microbial metagenomic analysis (before the phage analysis) that linked it to the clinical resolution? Again, discussion of this, bearing in mind the sequencing depth of these studies, would be helpful for the reader.

Thank for you this comment which was also brought up by Reviewer 1. Please see our responses to Reviewer 1 major concern point 3 and minor concern point 4. Briefly, we have toned down the language related to this finding and ensured that all references to it are labelled as putative as there is no confirmation of causality. Further, we have included a section in the limitations paragraph highlighting the importance of validating this finding experimentally and detailing why the bacterium may not have been detected as a marker of clinical resolution in the past. The new text reads:

“Moreover, the association between the taxon *Oscillospiraceae* bacterium CE91-St42 and its phage with clinical resolution following FMT remains putative and requires experimental validation to establish causality. *Oscillospiraceae* bacterium CE91-St42 was not identified to be associated with clinical resolution in past studies assessing the bacterial component of the microbiota[25,38,39]; however, this may have been a consequence of the isolate and its specific genome becoming available in 2022.”

The methods are clear and able to be followed.

Thank you. We appreciate the confirmation.

Project PRJEB26357 consists of 284 files metagenome. However, I was unable to find any metadata. The LOTUS data is unavailable, and the project number has not been provided. These two points make it impossible for anyone to repeat this work. Additionally, there is no statement that the authors agree to make this information available upon request.

We have updated our data availability statement to account for this. The new statement now reads:

“Shotgun metagenomic sequencing data from the FOCUS study has been previously submitted to the European Nucleotide Archive (ENA) under the accession number PRJEB26357. Shotgun metagenomic sequencing data from the LOTUS donors has been previously submitted to ENA under the accession number PRJEB50699. Shotgun metagenomic sequencing data from the LOTUS patients (PRJEB58035) as well as the long read sequencing data set (PRJEB76864) were generated as part of

this study and were submitted to ENA (currently set to private to be made publicly available on publication of the work). vOTU count tables and classifications for the FOCUS and LOTUS samples are available in Zenodo using DOI: 10.5281/zenodo.13627782 (currently embargoed and will be made publicly available on publication of the work). Patient metadata is available on request. Source data are provided with this paper.”

We thank the editor and reviewers for their kind consideration of our revised manuscript. We have addressed the remaining comments below.

Reviewer #1:

I would thank the authors for properly addressing the concerns of both my review and Reviewer #2.

I'm overall satisfied with the responses. I just want to add the below minor comments. You only need to address Q3. In the below I'll refer to the different comments as my initially major concerns Q1-Q4.

Thank you. Please find our responses below.

Q1: Thanks for addressing this. As I read the the procotol from the supplier it appear as sufficient for the purpose.

Q2: It is quite normal finding these eukaryotic viruses in fecal matter, so what you have done now is sufficient. It just have to be addressed.

The follow up to Q1 and Q2 are noted. Thank you.

Q3: Okay, I see its a fine argument in regard of the genetic overlap between the different vOTUs that likely are from the same phage. However, it would require long-read sequencing to do that like Nanopore sequencing, and in term of a more quantatively measurement, I would recommend you considering follow this paper: https://microbiomejournal.biomedcentral.com/articles/10.1186/s40168-018-0446-z - which is something you could add to the limitations as a future solution.

Thank you for this suggestion. We have now added this in the limitations section as a possible future direction. The additional text reads:

"Future work can consider optimized VLP purification and sequencing protocols that include exogenous phage spiking of fecal extracts for quantitative outputs.[37]"

The article is now cited as:
"37 Shkoporov, A. N., et al. Reproducible protocols for metagenomic analysis of human faecal phageomes. Microbiome 6, 68 (2018)."

Q4: Okay, fine with the limitations.

Thank you.

I wish you all the best luck in your future research.

Thank you for the time spent constructively reviewing our manuscript and the kind words. We appreciate it.

Reviewer #2:

I would like to thank the authors for their responses. The additional information they provided has improved the manuscript.

We appreciate the time spent constructively reviewing our manuscript. Thank you.
